# Transcriptome Analysis of Diurnal Gene Expression in Chinese Cabbage

**DOI:** 10.3390/genes10020130

**Published:** 2019-02-11

**Authors:** Jin A. Kim, Donghwan Shim, Shipra Kumari, Ha-eun Jung, Ki-Hong Jung, Heesu Jeong, Woe-Yeon Kim, Soo In Lee, Mi-Jeong Jeong

**Affiliations:** 1 Department of Agricultural Biotechnology, National Academy of Agricultural Science, Rural Development Administration, 370, Nongsaengmyeong-ro, Wansan-gu, Jeonju-si 560-500, Korea; tunnee.sipra@gmail.com (S.K.); jhe9135@naver.com (H.-e.J.); silee@korea.kr (S.I.L.); center1097@korea.kr (M.-J.J.); 2 Department of Forest Bio-resources, National Institute of Forest Science, Suwon 16631, Korea; shim.donghwan@gmail.com; 3 Department of Genetic Engineering and Crop Biotech Institute, Kyung Hee University, Yongin 17104, Korea; khjung2010@khu.ac.kr; 4 Department of Plant Science, College of Agriculture and Life Sciences, Seoul National University, San 56-1, Sillim-dong, Gwanak-gu, Seoul 151-744, Korea; mem0517@cnkgenomics.com; 5 Division of Applied Life Science (BK21Plus), Institute of Agricultural and Life Science (IALS), Gyeongsang National University, 501 Jinju-daero, Jinju 52828, Korea; kim1312@gnu.ac.kr

**Keywords:** *Arabidopsis*, *Brassica rapa*, circadian-related gene, polyploid genome, transcriptome

## Abstract

Plants have developed timing mechanisms that enable them to maintain synchrony with daily environmental events. These timing mechanisms, i.e., circadian clocks, include transcriptional/translational feedback loops that drive 24 h transcriptional rhythms, which underlie oscillations in protein abundance, thus mediating circadian rhythms of behavior, physiology, and metabolism. Circadian clock genes have been investigated in the diploid model plant *Arabidopsis thaliana*. Crop plants with polyploid genomes—such as *Brassica* species—have multiple copies of some clock-related genes. Over the last decade, numerous studies have been aimed at identifying and understanding the function of paralogous genes with conserved sequences, or those that diverged during evolution. *Brassica rapa’*s triplicate genomes retain sequence-level collinearity with *Arabidopsis*. In this study, we used RNA sequencing (RNAseq) to profile the diurnal transcriptome of *Brassica rapa* seedlings. We identified candidate paralogs of circadian clock-related genes and assessed their expression levels. These genes and their related traits that modulate the diurnal rhythm of gene expression contribute to the adaptation of crop cultivars. Our findings will contribute to the mechanistic study of circadian clock regulation inherent in polyploidy genome crops, which differ from those of model plants, and thus will be useful for future breeding studies using clock genes.

## 1. Introduction

Light is a primary energy source for plants—it regulates various stages of plant development and maintains high photosynthetic efficiency [1]. Sessile plants have developed timing mechanisms to maintain synchrony with the Earth’s rotation, and function according to daily environmental events, such as sunrise and sunset [2]. The subsequent transduction of environmental cues regulates, directly or indirectly, diverse developmental processes including photomorphogenesis, floral transition, leaf movements, stomatal conductance, photosynthetic capacity, volatile emissions [3,4,5], and interactions with abiotic and biotic factors [6,7]. These processes seem to have a diurnal rhythm, which occurs during daylight. Circadian rhythm is a biological process that behaves rhythmically during 24 h periods. Under diurnal conditions (light and temperature cycles), organismal genes are expressed due to free running circadian oscillations.

Recently, diurnally expressed genes have been identified in *Arabidopsis thaliana* [8]; the light signal transduction system—involving photo receptors—has been reported [9,10]; and a computational model of the plant clock system, comprising the main transcriptional feedback loop (loop I) and two additional associated loops (Loops II and III) [11], has been developed. High-throughput RNA sequencing (RNAseq) methods offer dramatic improvements over prior techniques [12,13]. RNA sequencing analysis of circadian transcriptomes derived from wild-type and period-null mutant (per0) *Drosophila* brains was performed. The results identified novel splicing events and transcripts regulated by the circadian clock. Furthermore, transcriptomic studies performed using several major crop plants including tomato, rice, polar, and lettuce, have demonstrated the expression of diurnal genes under environmental cycles and self-sustaining oscillated circadian genes under constant light or temperature [14,15,16].

The circadian clock coordinates physiological traits such as flowering time, photosynthesis, and growth, as well as internal metabolic and hormonal signals in plants with daily and seasonal environmental changes [17,18,19,20]. This coordination enhances plant fitness, growth, and environmental adaptation [21]. Clock-regulated genes are required for primary metabolism, including CO_2_ assimilation, starch accumulation/degradation in leaves, and nutrient (lipids and fatty acids) storage in developing seeds [18,20,22,23,24]. Additionally, potato tuberization and abiotic stress response-related secondary metabolic pathways are regulated by clock-related genes [25,26,27,28].

Crop plants that have undergone whole genome duplication events contain multiple copies of genes with or without functional redundancy [29,30,31,32,33,34,35,36,37]. Clock-related genes are conserved among angiosperm evolutionary lineages [33,38,39,40,41,42]. Although accumulation of genome sequences and comparative genome analysis techniques have accelerated the identification of circadian genes in polyploidy genomes, their functional specialization remains unclear. This raises interesting questions regarding how multiple members of gene families contribute to the circadian clock mechanism, and whether the clock model developed for *Arabidopsis* is applicable to plants with polyploid genomes. Functional studies involving paralogs improve our understanding of the unique physiology of each crop plant and establish a breeding strategy.

The polyploid genus *Brassica* is comprised of a variety of vegetable crops, such as Chinese cabbage, bok choy, turnip, and broccoletto, as well as oilseed crops, such as turnip rape and sarson, that are of worldwide agricultural importance. It is noteworthy that derived products such as processed oil and kimchi add economic value [43,44]. In the *B. rapa* genome, after multiple genome duplications and gene losses, circadian clock genes were preferentially retained relative to their neighboring genes [41,45]. Previous studies have described the structures of a pseudo-response regulator (PRR) and circadian clock-associated 1/late elongated hypocotyl (CCA1/LHY) gene families [40,41]. RNA sequencing reads from six tissues of *B. rapa* accession Chiifu-401-42—the genome of which has been sequenced—indicated widespread transcription of the Chinese cabbage genome [46]. We were interested in the role that the clock genes retained in the *B. rapa* genome play in the clock network, in addition to the developmental and metabolic processes regulated by them. Here we used RNAseq to profile the circadian transcriptome of leaves derived from wild-type *B. rapa*. Our findings will contribute to studies on the mechanisms of circadian clock regulation inherent in polyploidy genome crops—which differ from those of model plants—and to plant breeding strategies that require clock genes.

## 2. Materials and Methods

### 2.1. Plant Growth and Harvest

**Figure 1:**
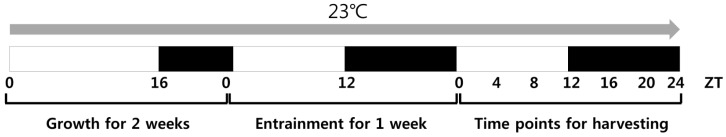
Chinese cabbage cultivation. Chinese cabbage seedlings were grown in a growth chamber in individual pots under a 16/8-h light/dark cycle for 2 weeks, with a cool-white fluorescent lamp. Plants were entrained for 1 week under a 12/12 h light/dark cycle. Aerial parts of three-week-old plants were harvested at ZT 0, 4, 8, 12, 16, 20, and 24 h, with two biological replicates. The plants were watered every three days, and the temperature was maintained at 23 °C until 3–5 true leaves emerged. White and black boxes indicate day (light on) and night (light off), respectively. ZT: zeitgeber time; ZT0: sunrise.

Chinese cabbage (*B. rapa* pekinensis) accession Chiifu-401-42 seeds were sown in individual pots containing soil and grown in a growth chamber (Eyelatron, FLI-301NH, Japan) under a 16/8 h light/dark cycle for 2 weeks, using a cool-white fluorescent lamp. Plants were entrained under a 12/12 h light/dark cycle for 1 week. The plants were watered every three days, and temperatures were maintained at 23 °C until 3–5 true leaves emerged. Aerial parts of three-week old plants were harvested at ZT (zeitgeber Time) 0, 4, 8, 12, 16, 20, and 24, with two biological repeats (Figure 1).

### 2.2. Library Preparation and RNA Sequencing

Total RNA was isolated from *B. rapa* leaf tissue using RNeasy Plant Mini Kits (Qiagen, Valencia, CA, USA), according to the manufacturer’s instructions. RNA quality was determined using a 2100 Bioanalyzer (Agilent, Santa Clara, CA, USA), and only samples with an RNA integrity number > 8 were used for library preparation. Preparation of each single-end complementary DNA (cDNA) library was conducted according to the TruSeq RNA Sample Preparation Guide (Illumina, San Diego, CA, USA). Complementary DNA libraries sequencing was performed using an Illumina HiSeq2000 sequencer. Base calls were made using CASAVA software (Illumina).

### 2.3. Transcript Quantification, Differential Expression Analysis, and Gene Annotation

Single-end reads were cleaned by processing with PRINSEQ-lite, v. 0.20.4 software (http://prinseq.sourceforge.net/). Sequences < 50 bp long, with at least one quality score < 10, a mean quality score of < 20, exact duplicates or reverse-complement exact duplicates were filtered out, and sequences with a quality score threshold of 20 were trimmed from both the 5′ and 3′ ends [47].

The clean reads of each sample were aligned to the reference transcriptome (B.rapa_197_transcript_primaryTranscriptOnly.fa of Phytozome V9.0, https://genome.jgi.doe.gov/portal/pages/dynamicOrganismDownload.jsf?organism=Phytozome) using Bowtie software [48]. RSEM v.1.3.0 software (http://deweylab.github.io/RSEM/) was used to generate read counts, and the trimmed mean of M-values (TMM)-normalized fragments per kb of exon per million (FPKM) reads mapped for each transcript [49]. For differential expression analysis, negative binomial dispersion across samples was calculated using the EdgeR v. 3.16.5 software [50]. Genes with more than a twofold change in expression, and a false discovery rate (FDR)-adjusted *p*-value ≤0.05, were considered to be differentially expressed. Information on gene annotation was supported by the *B.rapa*_197_annotation profile of Phytozome V9.0 (https://phytozome.jgi.doe.gov).

To identify rhythmically expressed genes, we applied the JTK-CYCLE circadian transcript analysis software. This is a non-parametric statistical algorithm designed to identify circadian-regulated transcripts and estimates their period, phase, and amplitude [51]. The dataset of *B. rapa* gene expression levels that we generated was used for the JTK-CYCLE analysis with a 24 h period. For comparison, we used the *Arabidopsis* microarray dataset from NCBI (https://www.ncbi.nlm.nih.gov/geo/query/acc.cgi?acc=GSE3416). Genes with a *q*-value < 0.05 were classified as rhythmic. Circadian-regulated genes from JTK-CYCLE were clustered using the MeV v.4.8.1 software [52]. Transcription factors (TFs) were classified into families using plant TF database (PlantTFDB) version 4.0 for TF annotation.

### 2.4. Quantitative Real-Time Polymerase Chain Reaction

Gene-specific primers were designed using Primer3 software (http://frodo.wi.mit.edu/primer3/) (Table S1). Three-week-old plants grown under a 12/12 h light/dark cycle, following growth under a 16/8 h light/dark cycle for 2 weeks, were used as input samples for qRT-PCR (quantitative real-time PCR). Plant tissues were collected at 4 h intervals, for a 24 h period under light/dark conditions, and for 72 h under continuous light conditions. Complementary DNA was synthesized using cDNA EcoDry Premix (Oligo dT) (Clontech, TaKaRa, Shiga, Japan). For cDNA synthesis, 5 ng of total RNA was incubated with oligo (dT) primers at 42 °C for 60 min, and heated at 70 °C for 10 min to stop the reaction. qRT PCR reactions were performed in three technical replicates using a Bio-Rad system (CFX Connect Real-Time PCR) and SYBR Green I Master Mix in a 20 µL final volume. Relative expression levels were determined as the expression ratio of target gene and β-actin in the same cDNA preparation.

### 2.5. Data Deposition

Raw read files were submitted to the sequence reads archive (SRA), NCBI database (accession number SRP166211).

## 3. Results

### 3.1. Sequencing and Statistical Evaluation of the Brassica rapa Transcriptome

To examine the transcriptome of Chinese cabbage (*B. rapa*, pekinensis), we entrained two-week-old seedlings under a 12/12 h light–dark cycle for 1 week. Two biological repeats for each sample were harvested at ZT 0, 4, 8, 12, 16, 20, and 24 h (Figure 1).

We isolated RNA from the aerial parts of the plants at seven time points and synthesized cDNA libraries for RNAseq, generating 1,363,558,142 raw reads. After filtering out low quality and unpaired reads, 1,363,198,067 high quality reads were used for mapping the *B. rapa* transcriptome. We obtained a mapping rate over 70% on the *B. rapa* reference transcript sequences (Table 1).

**Figure 2:**
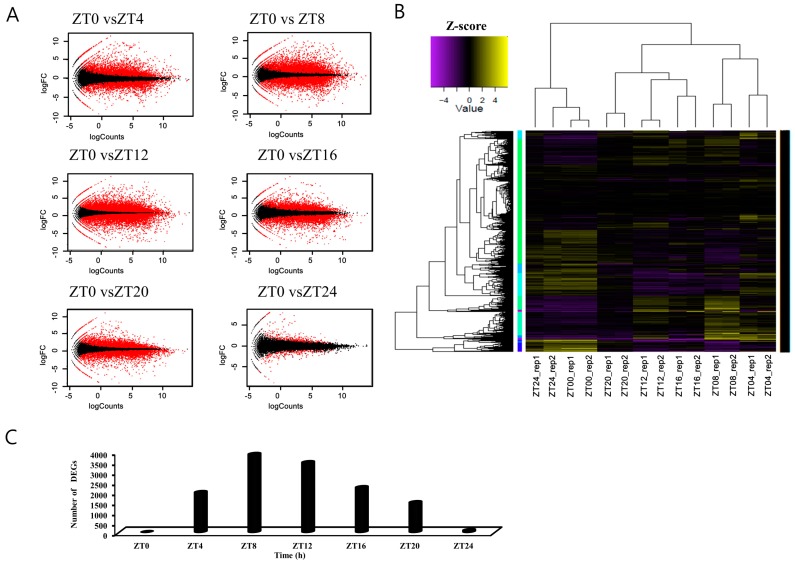
Statistical analysis of differentially expressed genes across samples. MA plots showing pairwise comparisons of transcript levels across samples. ZT0, 4, 8, 12, 16, 20, and 24 indicate time points after the light-on time. Y-axis: log2 fold change (logFC) between the two samples; X-axis: log2 average count normalized to the size factor. Red dots: transcripts with a logFC significantly higher than 2 or lower than −2; black dots: transcripts with a logFC of −2 to 2 (**A**). Clustered heatmap showing the Pearson correlation matrix for pairwise sample comparisons. The color key was adjusted based on the log2-centered values for optimal visual detection of differences, and the dendrograms show the distance between samples (**B**). The number of significantly expressed genes (FDR *p* <0.05) at each time point versus ZT0 (**C**).

To statistically evaluate the samples used for comparative transcriptomic analysis, we mapped high quality reads to the *B. rapa* (*Phytozome* v9.0) database and calculated transcript abundance. We identified differentially expressed genes (DEGs) based on pairwise sample comparisons with ZT0, and visualized DEGs distribution using MA (intensity ratio) plots (Figure 2A,C and Figure S2). In the pairwise ZT comparisons, each sample’s two biological replicates were highly correlated (range ± 1). ZT24 samples harvested during the same phase as the following day ZT0 were also highly correlated (Figure 2B and Figure S1).

### 3.2. Identification of Differentially Expressed Genes in Pairwise Sample Comparisons

In total, we detected 35,759 DEGs (87.2% of the 41,019 annotated genes) at one or more time points (using a cutoff of an at least twofold change and *p* <0.05, FDR *p*-value) in pairwise sample comparisons (Figure 2B). Initially, we compared the gene expression of each time point sample to ZT0 (Figure 2C). Among the DEGs, 1973, 3870, 3461, 2217, and 1471 putative transcripts were differentially expressed at ZT4, 8, 12, 16, and 20, respectively. Comparative analysis between ZT0 and ZT24 resulted in 96 DEGs (Figure 2C). After sunrise (ZT0), the number of DEGs was highest during the evening and at sunset (ZT8 and ZT12, respectively) and decreased sharply thereafter (Figure 2C).

### 3.3. Conserved Phasing of Diurnally Expressed Genes in *B. rapa*

**Figure 3:**
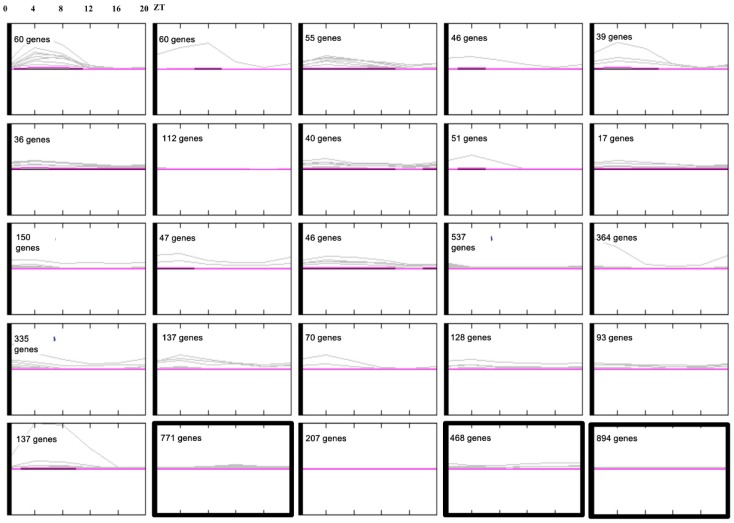
Clustering of 11,699 clock-regulated genes according to their diurnal cycle expression patterns in Chinese cabbage seedlings using JTK-CYCLE [51]. Thick boxes indicate groups that peaked during night time. Using JTK-CYCLE, 11,699 circadian-regulated genes were identified in leaves of Chinese cabbage seedlings growing under a 12/12 h light/dark cycle. These genes were clustered based on their diurnal expression. The k-means clustering was performed on fragments per kb of exon per million (FPKM) average values of the two repeats at 4 h intervals over 24 h using the MeV v. 4.8.1 software [52]. The number of genes in each cluster is shown.

Our results identified 11,699 of 41,019 putative unique transcripts (28.5%) in Chinese cabbage, and 5747 of 22,810 transcripts (25.2%) in *Arabidopsis* as circadian-regulated. Circadian-related transcripts were 3.3% more abundant in Chinese cabbage compared to *Arabidopsis* (Tables S3 and S4). Circadian-regulated genes clustered into 25 groups (Table S5). In the *B. rapa* dataset, transcripts with daytime expression peaks were classified into various groups, and those with nighttime peaks were classified into three groups (Figure 3). The phases of cycling genes were compared between *A. thaliana* and *B. rapa* datasets. Under diurnal conditions, most orthologs exhibited similar phases, but some showed an inverse expression (data not shown).

**Figure 4:**
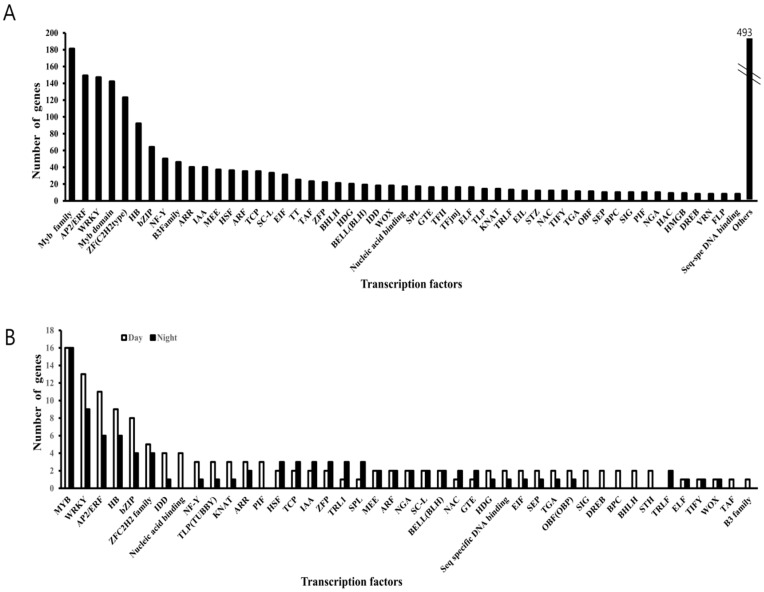
Classification of transcription factors (TFs) in the *B. rapa* transcriptome data set. (**A**) Total number of differentially expressed genes (DEGs) encoding TFs. (**B**) Forty-four transcription factors are circadian-regulated, where the white bar graphs represent genes upregulated in the daytime (ZR0–ZT12), and the black bar graph represents genes upregulated in the night time (ZR12–ZT24).

In the present study, 2,249 DEGs were identified as transcription factors (TFs). Functional annotation of these TFs based on their sequence homology and domain sequence similarities with the existing TF database of *B. rapa* resulting in 51 TF families which were over-represented (32.99% of total TFs); Myb family (181), AP2/ERF (149), WRKY (147), Myb domain (142), and ZF (C2H2 type) (123) TFs (Figure 4A). Among total TFs, 232 genes were circadian clock-regulated based on the DEGs pattern. MYB and WRKY TFs act as key regulators of many processes in plants, of which 16 MYB and 13 WRKY members appeared significantly upregulated during the daytime. The remaining 16 MYB and 9 WRKY members were downregulated during the daytime. Similarly, AP2/ERF, HB, bZIP, ZF (C2H2 type), IDD, NF-Y, TLP, PIF, and TCP are key regulators of plant growth and development. Of these families, 11, 9, 8, 5, 4, 3, 3, 3, and 2 members during daytime and 6, 6, 4, 4, 1, 1, 1, 0, and 3 during nighttime showed peaks of expression, respectively (Figure 4B). The rhythmic expression pattern suggests that these TFs may be related to the circadian function in *B. rapa*. Of the 51, forty four transcription factors have exhibited significant circadian enrichment (Figure 4B). TFs like Myb-related, basic leucine zipper (bZIP), and multiprotein bridging factor 1 (MBF1) were known associates of the circadian clock-regulated processes [53,54,55,56].

### 3.4. Expression Anaysis of Clock-Related Gene Paralogs in Chinese Cabbage

**Figure 5:**
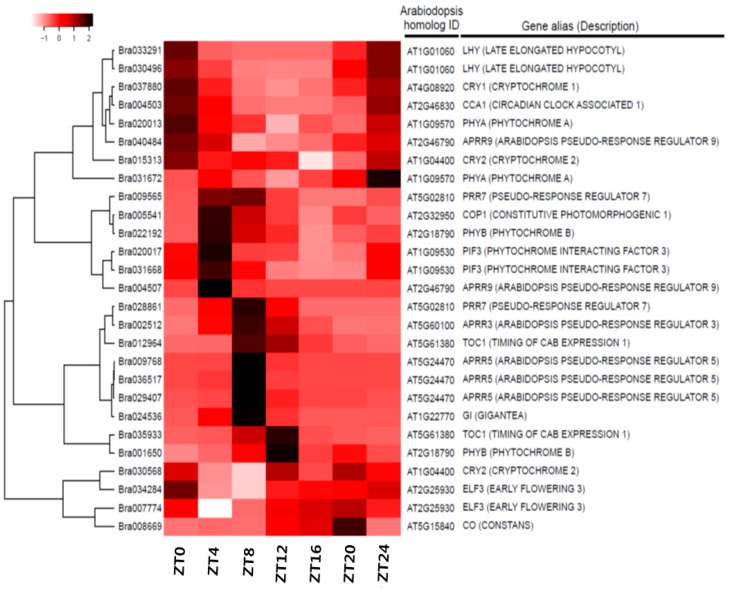
Expression of candidate clock-related genes over time in *B. rapa*. Heat map shows expression patterns of candidate clock-related genes. Candidate circadian-regulated genes have significantly different expression in *B. rapa* over time. The orthologs of *Arabidopsis* circadian-related genes identified based on the Kyoto Encyclopedia of Genes and Genomes (KEGG) pathways were screened in *B. rapa* (right). Dendrogram: hierarchical clustering of unigenes based on their expression pattern similarities. Gene ID numbers of the *B. rapa* sequence (Phytozome V9.0) (left). ZT0–24, time points after the light-on time (bottom).

Using *Arabidopsis* microarray datasets, we screened circadian pathway genes included in the Kyoto Encyclopedia of Genes and Genomes (KEGG) pathways (Figure S3) and determined their expression levels. Heat maps showed that the expression of these genes changed over time, and all exhibited clear peaks of expression except for *PHYA*, *COP1*, and *CRY2* (Figure S4). Based on a study of *Arabidopsis* clock regulation [57,58], we identified putative orthologs of the 21 key *Arabidopsis* clock-related genes (*CRY1*, *CRY2*, *PHYA*, *PHYB*, *COP1*, *PIF3*, *CCA1/LHY*, *GI*, *TOC1*, *ELF3*, *ELF4*, *RVE4*, *RVE8*, *LUX*, *FKF1*, *PRR1*, *PRR3*, *PRR5*, *PRR7*, and *PRR9*) present in the *B. rapa* dataset (Table 2 and Table S2) and confirmed daily fluctuations in their expression. One to three orthologous copies of individual *Arabidopsis* clock-related genes displayed highly conserved cycling profiles under diurnal conditions (Figure 5 and Figure S3). The expression cycling profiles of clock-related genes derived from the time course dataset showed similar expression patterns to those of *Arabidopsis* clock-related genes (Figure S4). The expression pattern of each ortholog resembled that of its corresponding gene in *Arabidopsis* with respect to, for example, the times at which their expression was highest and lowest (Figure 5 and Figure S4). However, some paralogous copies showed patterns, peak times, or intensities of expression that differed from their paralogs and their orthologs in *Arabidopsis*. For example, Bra030568—a *CRY2* ortholog—did not exhibit expression cycling, and the expression levels of Bra012964 and Bra035933—orthologs of *TOC1*—peaked at ZT8 and ZT12, respectively (Figure 5).

**Figure 6:**
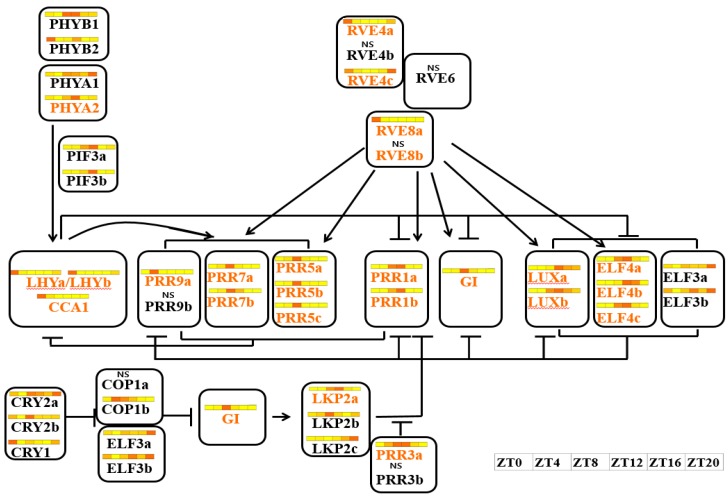
Circadian clock-related genes conserved in *B. rapa*. On the *Arabidopsis* clock network, all orthologous genes of *B. rapa* overlapped. The *Arabidopsis* clock network was adapted and modified from Lou et al. [41] and Hsu and Harmer [57]. Regulatory relationships in other networks are extrapolated from the *Arabidopsis* network rather than being derived empirically. Arrowheads and *t*-shaped arrows indicate positive and negative regulatory relationships, respectively. Yellow characters represent genes with free-running periods under continuous light. Colored tiles in bars indicate expression levels over 1 day. Expression levels decrease as the color changes from dark orange to white.

The RNAseq profiles and phase calls of the listed genes were estimated by calculating the FPKM values, and their expression was compared to their orthologs in *Arabidopsis* (Figure S5). Expression levels of Bra037880 and Bra024536, orthologs of cryptochrome circadian regulator 1 (*CRY1*) and *GI*, were highest and lowest at ZT12 and ZT8, respectively (Figure 6C,G). For Bra004503, a *CCA1* ortholog, expression levels were highest at ZT0 and lowest at ZT8-–ZT16 (Figure 5 and Figure S5O). Single copies of these genes were found for the *Arabidopsis* orthologs, and their expression patterns were highly conserved. Two homologous copies of phytochrome A (*PHYA*), phytochrome B (*PHYB*), constitutively photomorphogenic 1 (*COP1*), reveille 8 (*RVE8*), late elongated hypocotyl (*LHY*), and pseudo-response regulator (*PRR3*) were also found. One of the two paralogs (Bra020013, Bra022192, Bra005541, Bra029778, Bra033291, and Bra002512) showed expression patterns identical to their *Arabidopsis* orthologs (Figure 5 and Figure S5A,B,E,K,N,Q). However, Bra031672, Bra001650, Bra021818, Bra034074, Bra030496, and Bra020263 were rarely expressed. In *Arabidopsis*, *CRY2* expression did not exhibit a circadian pattern (Figures S4 and S5D). Our results also showed that the two copies of *CRY2* maintained steady expression over the course of 1 day (Figure 5 and Figure S5D). The timing of peak expression for the two paralogs of *ELF3*, *PRR1*, and *PRR7* differed. The expression of Bra007774 (Arabidopsis *ELF3*) peaked at ZT4, and that of Bra034284 peaked at ZT8 (Figure 5 and Figure S5H). Among the three *ELF4* homologs, the expression of Bra017035 peaked early, while Bra000165 and Bra004991 exhibited identical expression patterns (Figure 5 and Figure S5I). However, expression level peaks for the three homologs of *Arabidopsis ELF4* occurred at two different times (Figure 5 and Figure S5I). The *RVE4* homolog, Bra005754, was expressed at a low level with no diurnal pattern, but the expression of Bra005751 and Bra009562 began decreasing at sunrise, reached ZT8 at nadir, and subsequently increased after ZT16 (Figure 5 and Figure S5J). No homologs of *ZTL* or *FKF1* were found in the *B. rapa* transcriptome dataset (Table 2 and Table S2). Although each of these genes contains three functional domains—the LOV domain, the F-box motif, and the kelch repeats—and shows high sequence homology [59], only *LKP2* matched three homologous genes in our dataset. Bra038832, an *LKP2* homolog, showed a circadian expression pattern, but the expression levels of Bra038830 and Bra038831 remained steady over 1 day (Figure 5 and Figure S5M). Of the two *PRR1* homologs, Bra012964 expression peaked at ZT8. Furthermore, Bra035933 expression was lower than Bra012964 (Figure 5 and Figure S5P). The three *PRR5* homologs were expressed identical to that in *Arabidopsis*. However, Bra009768 expression peaked at ZT8 (Figure 5 and Figure S5R). The expression pattern of one Bra009565 homolog was identical to that of *Arabidopsis PRR7*, but that of Bra028861 peaked later at ZT8 (Figure 5 and Figure S5S). Bra033809 and Bra018204 expression peaked at ZT12, but that of the former was lower. The *PRR9* homolog, Bra004507, showed the same expression pattern as in *Arabidopsis*, but that of Bra040484 differed (Figure 5 and Figure S5T). We confirmed the free-running expression of all clock-related genes under continuous light for 72 h by quantitative reverse transcription-polymerase chain reaction (qRT-PCR) (Figure 6 and Figure S6). Expression of most orthologous copies of clock-related genes exhibited a cyclic pattern.

### 3.5. Differentially Expression of Glucosinolate-Related Gene Paralogs in Chinese Cabbage

**Figure 7:**
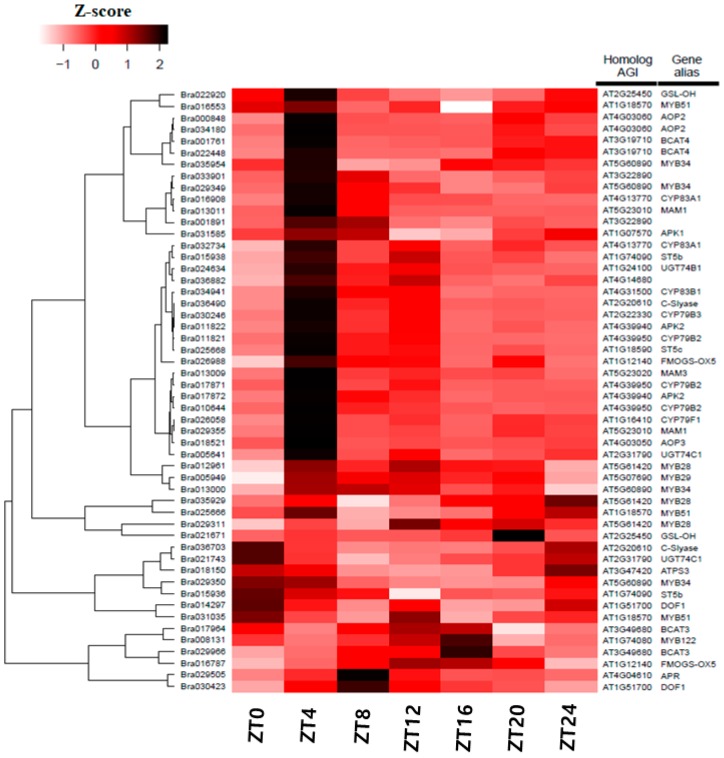
Expression pattern of glucosinolate-related genes in *B. rapa*. Heatmap shows significant differentially expressed candidate genes involved in the glucosinolate pathway in *B. rapa* [61]. Dendrogram: hierarchical clustering of unigenes based on their expression pattern similarities. Gene sequence IDs of the *B. rapa* (Phytozome V9.0) (left). ZT0–24: time points after the light-on time.

We identified orthologous genes related to the glucosinolate pathway in the *B. rapa* dataset. Expression of their paralogous genes as a heat map is shown in Figure 7. Though most genes were expressed during the daily cycle—at 4 h after sunrise (ZT4)—genes showed a peak expression. Some MYB TFs were also involved in indolic and aliphatic glucosinolate synthesis. Many Myb transcription factors, except MYB34s, showed peaks at different time points. Most paralogous members showed similar peak time points. Branched-chain aminotransferase (BCAT4) and methylthioalkylmalate synthase (MAM1) involved in the early steps of side chain elongation, and two AOP2 and AOP3 responsible for the side chain modification step in the glucosinolate pathway [60] had peak expression at ZT4. We found that three sulpho-transferases (ST) were involved in the core structure of glucosinolate biosynthesis. Among them, one *ST5b* (Bra015938) and *ST5c* (Bra025668) showed high expression at ZT4, and the other *ST5b* (Bra015936) at ZT0. Expression level then gradually decreased during a 24 h period. Similarly, one *FMOGS-OX5* (Bra026988) showed peak expression at ZT4 but the other (Bra016787) at ZT16.

Additionally, other genes such as one *DOF1* (Bra014297) had peak expression at ZT0, while others were at ZT8 (Bra030423), ZT4 during the light period (one *GSL-OH* (Bra022920)), and at ZT20 during the dark period (Bra021671). All *CYP* genes like *CYP79B2*, *CYP79B3*, *CYP79F1*, *CYP83A1*, *CYP83B1*, and *CYP83B2* were highly expressed at ZT4 and showed expression throughout the 24 h cycle.

## 4. Discussion

Photocycles drive oscillations in gene expression. At least 10–30% of genes expressed in *Arabidopsis* are regulated in a circadian manner [21,62], and up to 89% of transcripts in *Arabidopsis* display rhythmic expression patterns under a broad set of environmental diurnal/circadian conditions. Indeed, approximately 60% of rice and poplar transcripts displayed rhythmic expression under diurnal/circadian conditions [16].

### 4.1. Circadian-Regulated Genes in the Brassica Genome

The *Brassica* genome underwent a whole-genome triplication event after divergence from *A. thaliana* [45], in addition to several very recent genome duplications. These events make the genus Brassica useful for studies of polyploid genomes’ evolution. The *B. rapa* genome, which was the first to be sequenced among the *Brassica* species [45], is 529 Mbp with an approximately 220 Mbp euchromatic region (42% of the genome) [36]. In this study, 28.5% of the 41,019 putative unique transcripts in *B. rapa* were estimated to be circadian-regulated. These findings are similar to those in *Arabidopsis* [63], in which 25% of 22,811 transcripts were circadian-regulated. These data suggest that the number of genes whose expression shows a daily cycle and the total number of genes in the Chinese cabbage genome are twofold those in *Arabidopsis*. In seedling leaf tissues, 35,759 genes (87% of the total of 41,020 annotated genes) were expressed at more than one time point. This is higher than the 32,335 genes (78.8% of the 41,020 annotated genes) expressed in at least one tissue of *B. rapa* Chiifu [46]. These results suggest that the expression of many genes undergo a daily cycle. In plants, many TFs act as key regulators of various plant functions such as cell cycle, metabolic and physiological pathways, and responses to the environment. More than 2000 TFs have been found in the Arabidopsis genome [64,65,66,67]. Circadian clocks, as timekeeping systems, generate endogenous rhythms with periods of about a day. In the core system based on a transcriptional–translational negative feedback loop, CCA1 and LHY encode closely related single MYB domain transcription factors [68,69]. In addition, various TFs are involved in the regulation of light signal transduction and circadian rhythm [70]. Among the 2269 TFs found in the *B. rapa* dataset, approximately 14% from each group were found to have a daily cycle of expression (Figure 4B). In Arabidopsis, similar TF genes are regulated by blue light [71]. Nine transcription factor families in Arabidopsis, including NAC, bHLH, C2H2, AP2/ERF, the MYB superfamily, HB, WRKY, bZIP, and MADS are either induced or repressed by blue light [71].

### 4.2. Circadian Genes Conserved in Brassica after Genome Duplication Events

After triploidization, *B. rapa* genome segments retained 36–70% of their original genes during fractionation [45]. At the genomic level, approximately double the number of *Arabidopsis* genes were maintained due to genome shrinkage in the *B. rapa* genome [36]. After triplication and fractionation, circadian clock-related genes were retained in *B. rapa* at a significantly higher rate than other gene sets. Furthermore, 65% of the clock-related genes were retained more than one copy—two or three [72]. Our findings are in agreement with previously published findings (Table 2). Two single myeloblastosis (Myb) domain transcription factors play critical roles in the central loop of the *Arabidopsis* clock [11,73]. The orthologs of *LHY/CCA1* are found as three gene family members in *B. rapa*. One *LHY* and *CCA1* orthologs (*Bra033291 and Bra004503*) were similarly expressed as those in *Arabidopsis*, but the expression of Bra030496 was lower in *B. rapa* (Figure 5 and Figure S5N,O). Among the 18 *RVE* homologs with roles in clock function or regulation of clock output pathways in *Arabidopsis* [56,74,75,76,77], our transcriptome dataset contained three and two orthologs of *REV4* and *REV8*, respectively (Table 2). The expression pattern of each was similar to that of its ortholog in *Arabidopsis*, but the expression of orthologs of *REV4* and *REV8*, Bra005754 and Bra034074, was lower (Figure 5 and Figure S5J,K).

### 4.3. Expression Patterns of Paralogous Gene Copies

*Pseudo-Response Regulator* gene, found in the moss *Physcomitrella patens*, has been generated very early in evolution [78]. After its divergence from *Carica papaya*, only a single copy of each *PRR* gene was retained in *Arabidopsis*. However, after genome triplication in *B. rapa*, earlier work based on microsynteny analysis estimated a significantly higher rate of retention than that of their neighboring genes [41]. Eleven *PRR* genes were identified in the *B. rapa* genome. *PRR3b* and *PRR9b* detected as a result of rearrangements and partial deletions were predicted to be nonfunctional or neo-functional [41]. We confirmed the rare expression of *PRR3b*, with very low FPKM values and no specific qRT-PCR evidence (Figure 5 and Figure S5Q). Although it was not detected by qRT-PCR (Figure 5 and Figure S5Q), RNAseq showed that *PRR9b* expression was higher than *PRR9a* (Figure 5 and Figure S5T). *PRR3* modulates the stability of *TOC1* (*PRR1*) through a direct protein–protein interaction [79]. In the *B. rapa* genome, the expression of two *PRR1* orthologs peaked at different time points (Table 2 and Figure S5P), suggesting that the circadian clock in *B. rapa* is regulated by a novel mechanism.

In *Arabidopsis*, *ZTL* and its close relatives *LKP2* and *FKF1* determine the length of the circadian period by regulating the degradation of *TOC1* [80,81]. These three genes encode F-box proteins with an N-terminal LOV domain, a central F-box, and a series of Kelch repeats at the C-terminus [41,59]. Although their sequences are similar, *ZTL* and *FKF1* were not found and three tightly linked copies of *Arabidopsis LKP2* were found in *B. rapa* [41]. Based on their domain structures, *FKF1* in *Arabidopsis* has one less repeat (four) compared to *ZTL* and *LKP2* (five each). In *B. rapa*, *LKP2a* (Bra038832) has one less repeat (four) compared to *LKP2b* (Bra038831) and *LKP2c* (Bra038830) (five each) [41]. Interestingly, the expression of *LKP2a* (Bra038832) peaked at around ZT8, similar to *Arabidopsis FKF1* (Figures S5M,U,V and S6). *LKP2b* and *LKP2c* did not show a rhythmic expression pattern. *Arabidopsis ZTL*, *LKP2*, and *FKF1* play overlapping roles in circadian period determination. Although *ZTL* predominates, *FKF1* plays the major role in flowering time determination [80,81,82]. The functional specialization of the three copies of *LKP2* in *B. rapa* warrants further research. Homologous genes with multiple copies are considered to be functionally similar. Orthologous copies have redundant function and may show functional specialization or sub-functionalization compared to the ancestral copy [83]. However, one or more members may acquire new functions, while others retain their original function [84,85,86,87,88]. Such changes may be advantageous for preserving fitness. We found different expression levels or phase shifts among paralogous copies (Figure S5). Further research on the function of each paralogous copy is needed to explain the circadian rhythm of gene expression in crop species.

### 4.4. Regulation of Circadian-Mediated Biological Processes in Crops

The understanding of the circadian clock and its regulatory processes can be effectively utilized to enhance plant growth and fitness [20,57,89,90]. The synthesis of glucosinolates, a major secondary metabolite in *B. rapa,* has been reported as a light-regulated pathway. In the first 4 h of re-illumination of dark-adapted plants, the expression of many glucosinolate genes increased, inducing accumulation of mRNA [91]. BCAT4 and MAM1 are involved in the early side chain elongation process, and AOP2 in the secondary modification of the side chain, which showed higher expression levels under the light condition compared to darkness. They maintained high expression levels when the plants were kept under continuous light [60,91]. Reciprocally, glucosinolate genotypes altered the periodicity of a clock output unrelated to glucosinolates, the photochemical state of photosystem II [92]. These findings suggest that the manipulation of circadian clock genes can be valuable for improving plant metabolism. Studies on the expression patterns of clock-related genes in crop species will enable the formulation of effective crop-breeding strategies.

## Figures and Tables

**Table 1:** Statistical analysis of *Brassica rapa* RNA sequencing (RNAseq) reads. ZT: zeitgeber Time.

Index	Sample Name	Num. of Raw Reads	Num. of Cleaned Reads	Mean Length of Cleaned Reads (bp)	Num. of Mapped Reads *to Brassica rapa* Transcripts	Percent of Mapped Reads
1	ZT0_rep1	102,435,614	102,408,172	98.25	73,819,707	72.08%
2	ZT0_rep2	96,521,352	96,496,334	98.28	69,622,768	72.15%
3	ZT4_rep1	87,345,158	87,321,395	98.13	63,413,638	72.62%
4	ZT4_rep2	100,754,314	100,727,654	98.21	73,690,375	73.16%
5	ZT8_rep1	97,511,686	97,485,539	98.24	69,267,392	71.05%
6	ZT8_rep2	96,598,065	96,572,858	98.23	70,045,246	72.53%
7	ZT12_rep1	95,002,635	94,977,251	98.25	69,348,349	73.02%
8	ZT12_rep2	96,254,940	96,229,049	98.28	70,228,180	72.98%
9	ZT16_rep1	92,587,264	92,562,453	98.27	66,796,081	72.16%
10	ZT16_rep2	96,368,659	96,342,993	98.25	68,985,090	71.6%
11	ZT20_rep1	98,509,742	98,483,494	98.25	70,150,697	71.23%
12	ZT20_rep2	94,190,011	94,164,614	98.29	67,805,050	72.01%
13	ZT24_rep1	100,934,518	100,909,158	98.26	72,773,028	72.12%
14	ZT24_rep2	108,544,184	108,517,103	98.28	77,988,632	71.87%

**Table 2:** The lists of orthologous genes included in circadian clock pathway.

AGI	Alias	Description	ID	PER	LAG	AMP
AT1G09570.1	FHY2,FRE1,HY8,PHYA	phytochrome A	Bra020013	24.00	2.00	3.57
			Bra031672	18.00	NA	0.06
AT2G18790.1	HY3,OOP1,PHYB	phytochrome B	Bra001650	24.00	13.00	0.07
			Bra022192	22.00	7.15	3.58
AT4G08920.1	ATCRY1,BLU1,CRY1,HY4,OOP2	cryptochrome 1	Bra037880	24.00	2.00	58.64
AT1G04400.1	AT-PHH1,ATCRY2,CRY2,FHA,PHH1	cryptochrome 2	Bra015313	12.00	0.00	9.70
			Bra030568	10.00	NA	0.00
AT2G32950.1	ATCOP1,COP1,DET340,EMB168,FUS1	transducin/WD40 repeat-like superfamily protein	Bra005541	20.00	7.00	5.27
			Bra021818	18.00	NA	0.04
AT1G09530.1	PAP3,PIF3,POC1	phytochrome interacting factor 3	Bra020017	24.00	5.00	3.75
			Bra031668	24.00	5.00	2.83
AT1G22770.1	FB,GI	gigantea protein (GI)	Bra024536	24.00	8.00	8.20
AT2G25930.1	ELF3,PYK20	hydroxyproline-rich glycoprotein family protein	Bra007774	24.00	18.00	4.14
			Bra034284	20.00	0.00	2.22
AT2G40080.1	ELF4	Protein of unknown function (DUF1313)	Bra000165	24.00	13.00	13.00
			Bra004991	24.00	14.00	19.58
			Bra017035	20.80	12.58	20.58
AT5G02840.1	LCL1(RVE4)	LHY/CCA1-like 1	Bra005751	24.00	0.00	14.20
			Bra005754	22.00	19.07	0.10
			Bra009562	22.00	0.00	10.42
AT3G09600.1	RVE8	homeodomain-like superfamily protein	Bra029778	24.00	2.00	19.94
			Bra034074	24.00	2.00	10.72
AT3G46640.1	LUX,PCL1	homeodomain-like superfamily protein	Bra018204	24.00	16.00	4.89
			Bra033809	22.00	14.12	8.36
AT2G18915.2	ADO2,LKP2	LOV KELCH protein 2	Bra038830	24.00	3.00	1.58
			Bra038831	24.00	2.00	1.29
			Bra038832	22.67	11.96	5.81
AT1G01060.1 AT1G01060.4	LHY,LHY1	homeodomain-like superfamily protein	Bra030496	24.00	1.00	34.47
			Bra033291	24.00	0.00	37.84
AT2G46830.1	CCA1	circadian clock associated 1	Bra004503	24.00	2.00	33.20
AT5G61380.1	APRR1,AtTOC1,PRR1,TOC1	CCT motif -containing response regulator protein	Bra012964	22.67	11.96	15.09
			Bra035933	24.00	12.00	6.58
AT5G60100.1 AT5G60100.2	APRR3,PRR3	pseudo-response regulator 3	Bra002512	20.00	10.00	22.85
			Bra020263	18.00	NA	NA
AT5G24470.1	APRR5,PRR5	pseudo-response regulator 5	Bra009768	20.00	10.00	1.19
			Bra029407	20.00	10.00	3.28
			Bra036517	20.00	8.00	2.12
AT5G02810.1	APRR7,PRR7	pseudo-response regulator 7	Bra009565	24.00	8.00	25.25
			Bra028861	22.00	10.08	40.83
AT2G46790.1	APRR9,PRR9,TL1	pseudo-response regulator 9	Bra004507	24.00	6.00	0.11
			Bra040484	22.67	0.62	12.55

PER: period; LAG: peak time; AMP: amplitude from JCK-CYCLE.

## Supplementary Materials

The following are available online at https://www.mdpi.com/2073-4425/10/2/130/s1. Figure S1: Statistical analysis of differentially expressed genes across repeat samples; Figure S2: Statistical analysis of significantly expressed genes at each time point versus ZT0; Figure S3: Circadian-related genes in *Arabidopsis* and *B. rapa*.; Figure S4: Expression of clock-related genes over time in *Arabidopsis* and PCC values between Br and At; Figure S5: Expression of clock-related genes in *Arabidopsis* and *B. rapa*. Bra numbers indicate orthologs of *PHYA* (A), *PHYB* (B), *CRY1* (C), *CRY2* (D), *COP1* (E), *PIF3* (F), *GI* (G), *ELF3* (H), *ELF4* (I), *RVE4* (J), *RVE8* (K), *LUX* (L), *LKP2* (M), *LHY* (N), *CCA1* (O), *PRR1* (P), *PRR3* (Q), *PRR5* (R), *PRR7* (S), *PRR9* (T), *ZTL*(U), and *FKF1*(V); Figure S6: Free-running expression of clock-related genes in *B. rapa*; Table S1: qRT-PCR primers used in this work.; Table S2: *B. rapa*_circadian_normalized_FPKM and orthologous identification; Table S3: JTK-cycle results of the *Arabidopsis* data set; Table S4: JTK-cycle results of the *B. rapa* data set; Table S5: Clustering results of cycling genes in *B. rapa*.
