# Supplementary material for: Transcriptome Analysis of Diurnal Gene Expression in Chinese Cabbage
Source: Genes (Basel). 2019 Feb 11;10(2):130. doi: 10.3390/genes10020130 (PMC6409912; doi:10.3390/genes10020130)
Supplement: Supplementary file 1 [file genes-10-00130-s001.zip › supplement fig_ver1.pptx]

## Slide 1
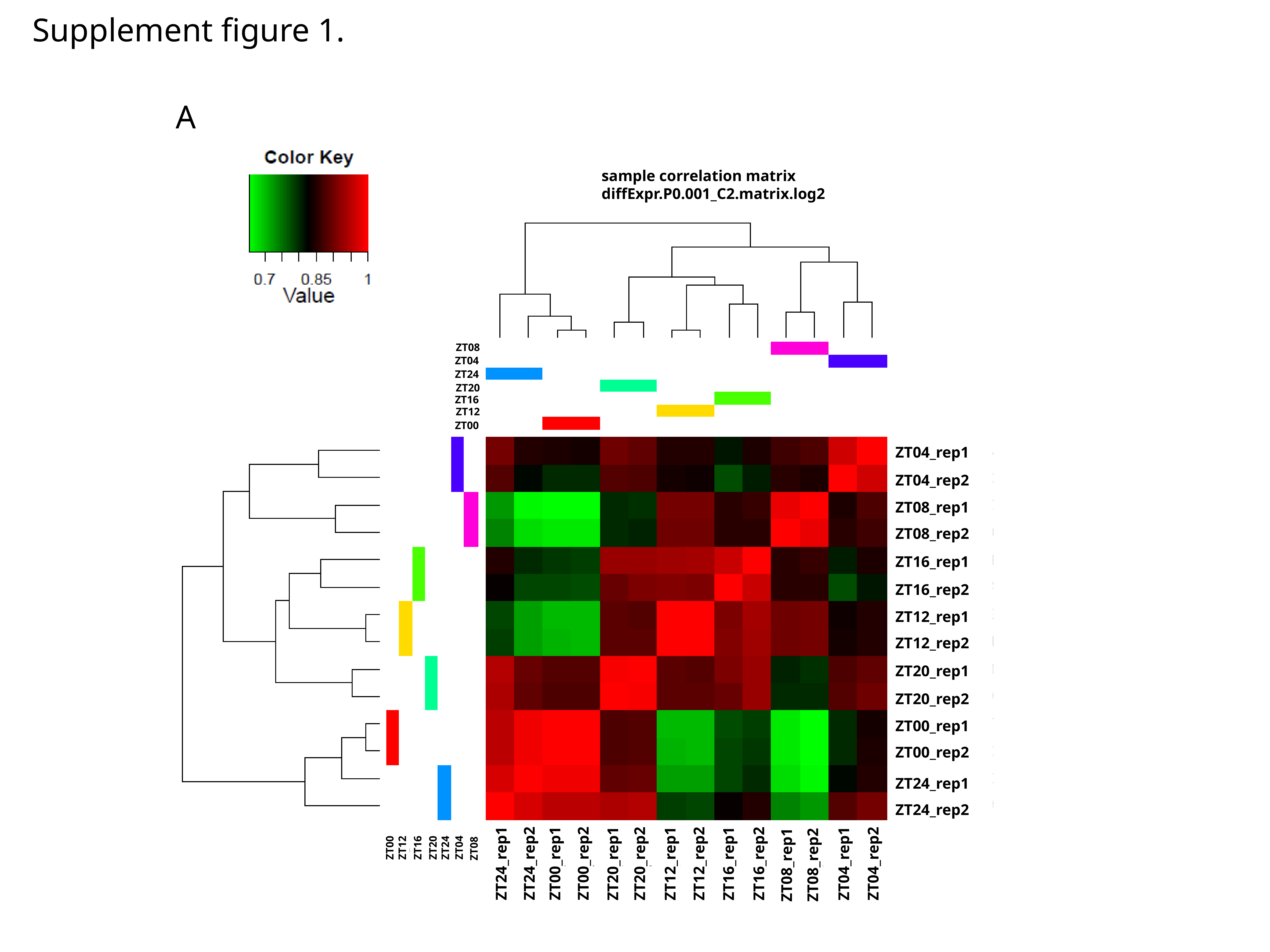

Supplement figure 1.
A
ZT04_rep1
ZT04_rep2
ZT08_rep1
ZT08_rep2
ZT16_rep1
ZT16_rep2
ZT12_rep1
ZT12_rep2
ZT20_rep1
ZT20_rep2
ZT00_rep1
ZT00_rep2
ZT24_rep1
ZT24_rep2
ZT24_rep2
ZT00_rep2
ZT24_rep1
ZT00_rep1
ZT20_rep1
ZT16_rep1
ZT16_rep2
ZT04_rep1
ZT04_rep2
ZT20_rep2
ZT12_rep1
ZT12_rep2
ZT08_rep1
ZT08_rep2
sample correlation matrix
diffExpr.P0.001_C2.matrix.log2
ZT08
ZT04
ZT24
ZT20
ZT16
ZT12
ZT00
ZT04
ZT00
ZT12
ZT16
ZT20
ZT24
ZT08

## Slide 2
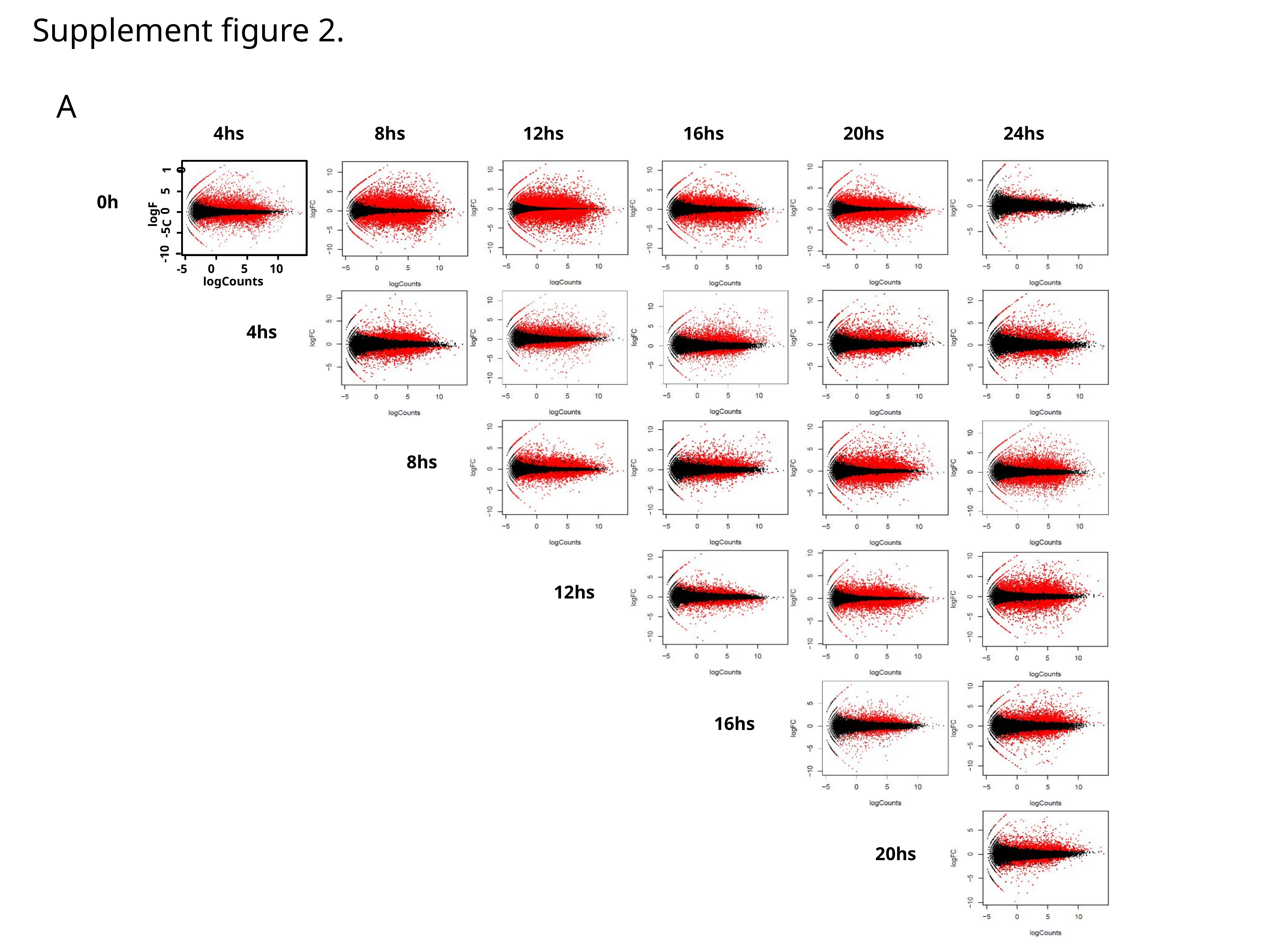

Supplement figure 2.
A
4hs
8hs
12hs
16hs
20hs
24hs
0h
4hs
8hs
12hs
16hs
20hs
10
5
logFC
0
-5
-10
0
5
10
-5
logCounts

## Slide 3
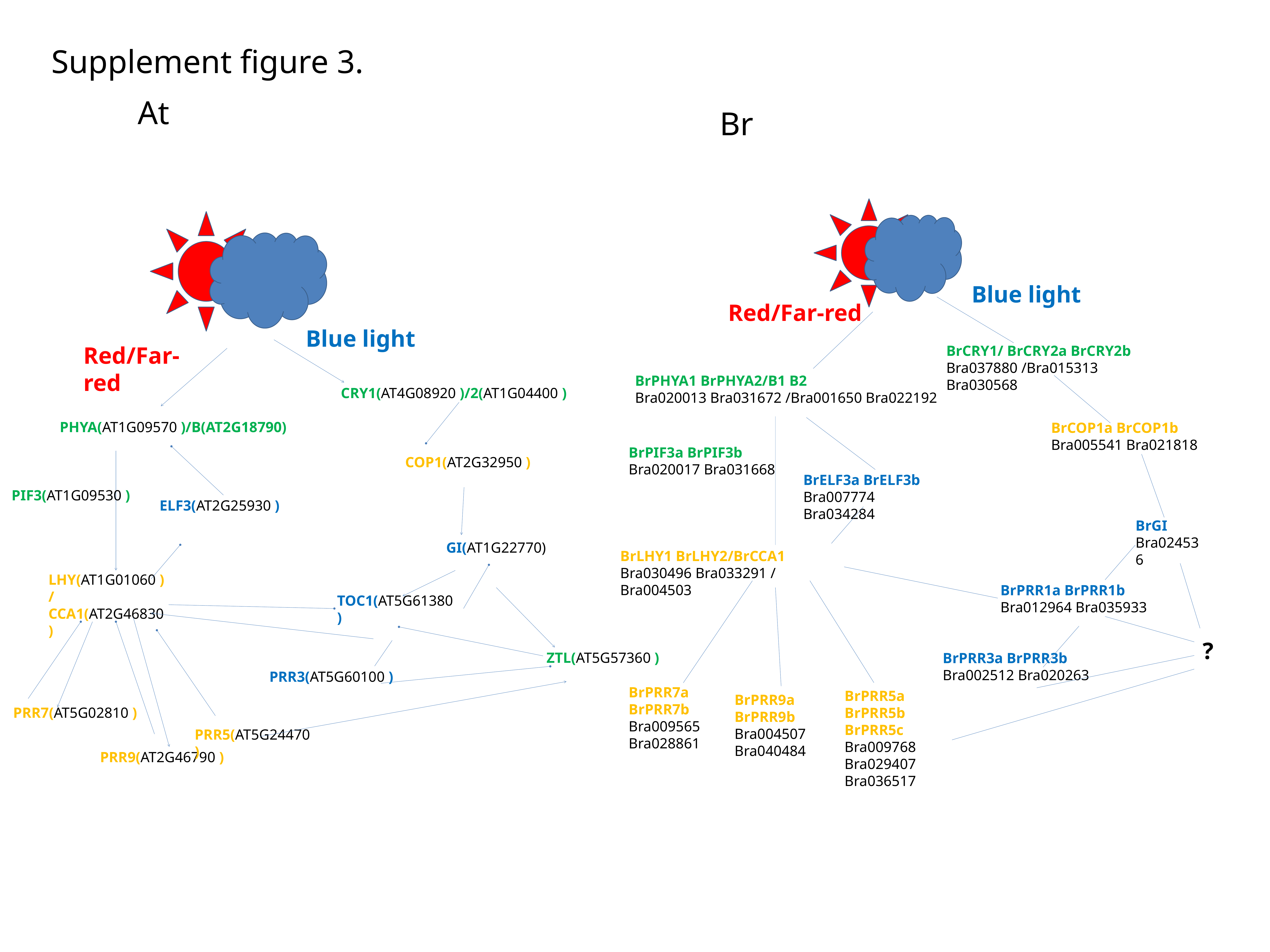

Supplement figure 3.
At
Br
Blue light
Red/Far-red
BrCRY1/ BrCRY2a BrCRY2b
Bra037880 /Bra015313 Bra030568
BrPHYA1 BrPHYA2/B1 B2
Bra020013 Bra031672 /Bra001650 Bra022192
BrCOP1a BrCOP1b
Bra005541 Bra021818
BrPIF3a BrPIF3b
Bra020017 Bra031668
BrELF3a BrELF3b
Bra007774 Bra034284
BrGI
Bra024536
BrLHY1 BrLHY2/BrCCA1
Bra030496 Bra033291 /Bra004503
BrPRR1a BrPRR1b
Bra012964 Bra035933
?
BrPRR3a BrPRR3b
Bra002512 Bra020263
BrPRR7a
BrPRR7b
Bra009565 Bra028861
BrPRR5a
BrPRR5b
BrPRR5c
Bra009768 Bra029407 Bra036517
BrPRR9a
BrPRR9b
Bra004507 Bra040484
Blue light
Red/Far-red
CRY1(AT4G08920 )/2(AT1G04400 )
PHYA(AT1G09570 )/B(AT2G18790)
COP1(AT2G32950 )
PIF3(AT1G09530 )
ELF3(AT2G25930 )
GI(AT1G22770)
LHY(AT1G01060 )/CCA1(AT2G46830 )
TOC1(AT5G61380 )
ZTL(AT5G57360 )
PRR3(AT5G60100 )
PRR7(AT5G02810 )
PRR5(AT5G24470 )
PRR9(AT2G46790 )

## Slide 4
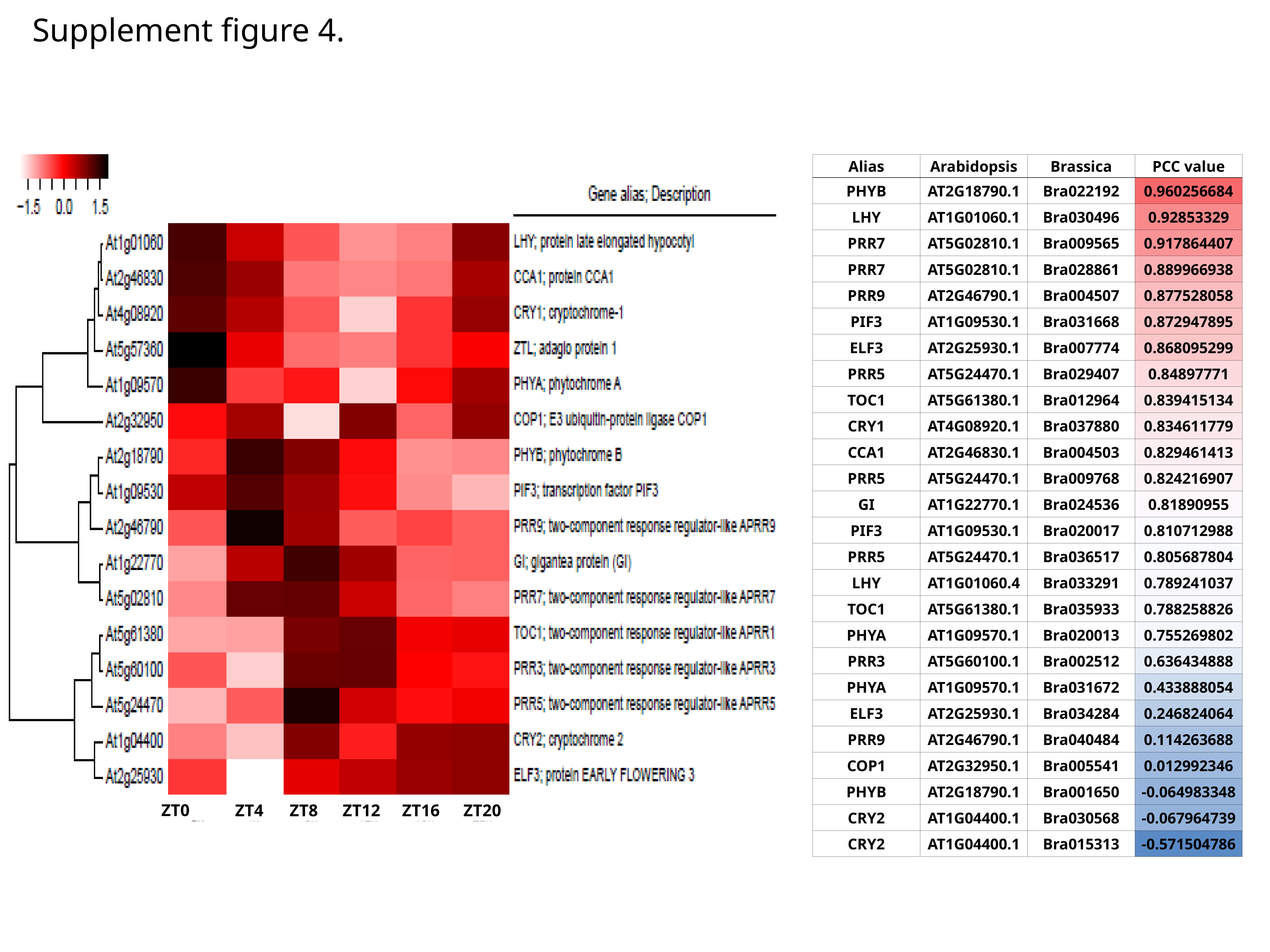

Supplement figure 4.
| Alias | Arabidopsis | Brassica | PCC value |
| --- | --- | --- | --- |
| PHYB | AT2G18790.1 | Bra022192 | 0.960256684 |
| LHY | AT1G01060.1 | Bra030496 | 0.92853329 |
| PRR7 | AT5G02810.1 | Bra009565 | 0.917864407 |
| PRR7 | AT5G02810.1 | Bra028861 | 0.889966938 |
| PRR9 | AT2G46790.1 | Bra004507 | 0.877528058 |
| PIF3 | AT1G09530.1 | Bra031668 | 0.872947895 |
| ELF3 | AT2G25930.1 | Bra007774 | 0.868095299 |
| PRR5 | AT5G24470.1 | Bra029407 | 0.84897771 |
| TOC1 | AT5G61380.1 | Bra012964 | 0.839415134 |
| CRY1 | AT4G08920.1 | Bra037880 | 0.834611779 |
| CCA1 | AT2G46830.1 | Bra004503 | 0.829461413 |
| PRR5 | AT5G24470.1 | Bra009768 | 0.824216907 |
| GI | AT1G22770.1 | Bra024536 | 0.81890955 |
| PIF3 | AT1G09530.1 | Bra020017 | 0.810712988 |
| PRR5 | AT5G24470.1 | Bra036517 | 0.805687804 |
| LHY | AT1G01060.4 | Bra033291 | 0.789241037 |
| TOC1 | AT5G61380.1 | Bra035933 | 0.788258826 |
| PHYA | AT1G09570.1 | Bra020013 | 0.755269802 |
| PRR3 | AT5G60100.1 | Bra002512 | 0.636434888 |
| PHYA | AT1G09570.1 | Bra031672 | 0.433888054 |
| ELF3 | AT2G25930.1 | Bra034284 | 0.246824064 |
| PRR9 | AT2G46790.1 | Bra040484 | 0.114263688 |
| COP1 | AT2G32950.1 | Bra005541 | 0.012992346 |
| PHYB | AT2G18790.1 | Bra001650 | -0.064983348 |
| CRY2 | AT1G04400.1 | Bra030568 | -0.067964739 |
| CRY2 | AT1G04400.1 | Bra015313 | -0.571504786 |
ZT0
ZT4
ZT8
ZT12
ZT16
ZT20

## Slide 5
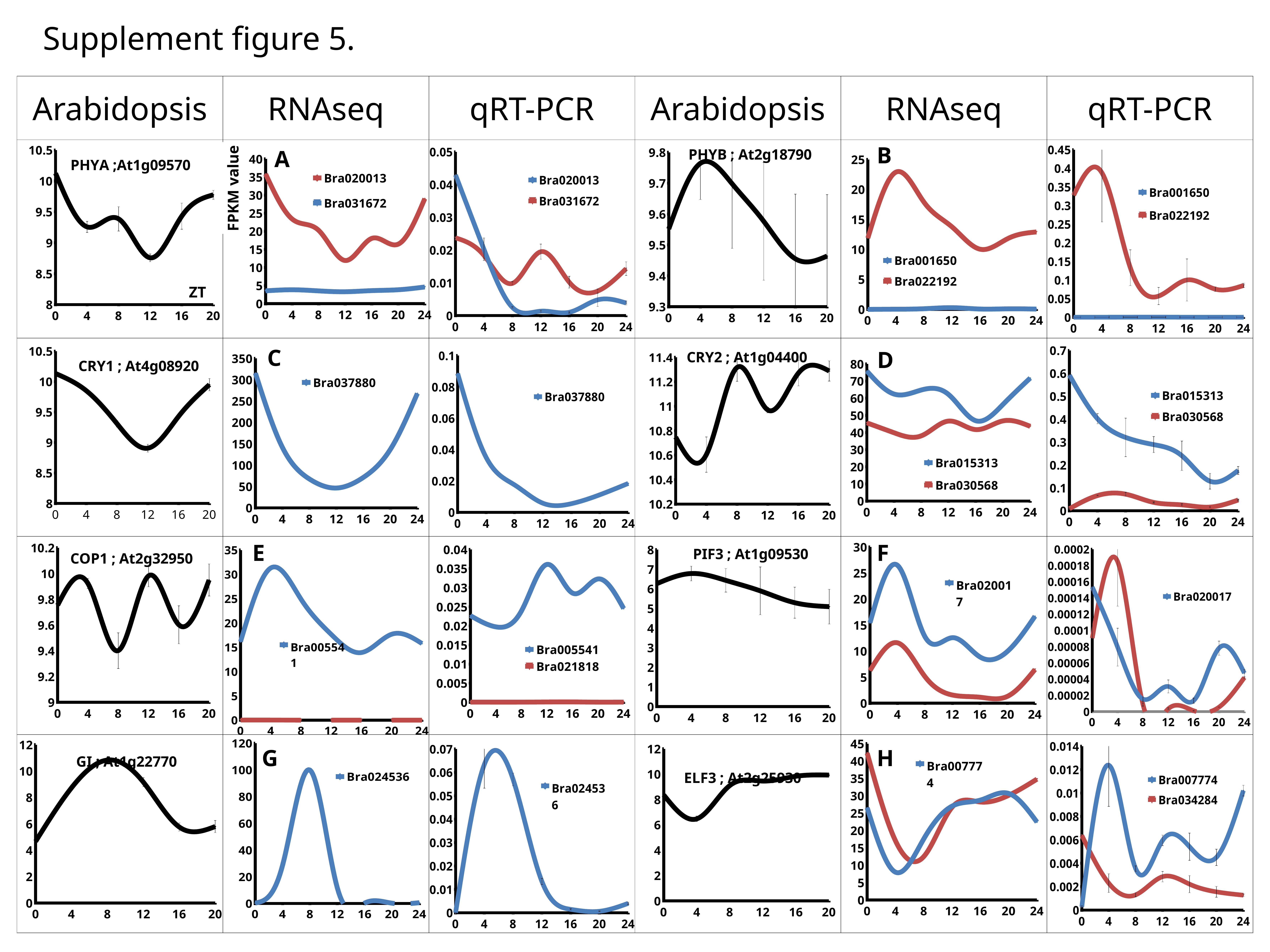

Supplement figure 5.
| Arabidopsis | RNAseq | qRT-PCR | Arabidopsis | RNAseq | qRT-PCR |
| --- | --- | --- | --- | --- | --- |
| | | | | | |
| | | | | | |
| | | | | | |
| | | | | | |
### Chart: PHYA ;At1g09570
| Category | PHYA At1g09570 |
|---|---|
### Chart
| Category | Bra020013 | Bra031672 |
|---|---|---|
### Chart: PHYB ; At2g18790
| Category | PHYB At2g18790 |
|---|---|B
### Chart
| Category | Bra001650 | Bra022192 |
|---|---|---|
### Chart
| Category | Bra020013 | Bra031672 |
|---|---|---|
### Chart
| Category | Bra001650 | Bra022192 |
|---|---|---|FPKM value
ZT
### Chart: CRY1 ; At4g08920
| Category | CRY1 At4g08920 |
|---|---|
### Chart
| Category | Bra037880 |
|---|---|
### Chart
| Category | Bra037880 |
|---|---|
### Chart
| Category | Bra015313 | Bra030568 |
|---|---|---|
### Chart
| Category | Bra015313 | Bra030568 |
|---|---|---|
### Chart: CRY2 ; At1g04400
| Category | CRY2 At1g04400 |
|---|---|
### Chart: COP1 ; At2g32950
| Category | COP1 At2g32950 |
|---|---|
### Chart
| Category | Bra005541 | Bra021818 |
|---|---|---|E
### Chart
| Category | Bra005541 | Bra021818 |
|---|---|---|
### Chart
| Category | Bra020017 | Bra031668 |
|---|---|---|F
### Chart: PIF3 ; At1g09530
| Category | PIF3 At1g09530 |
|---|---|
### Chart
| Category | Bra020017 | Bra031668 |
|---|---|---|
### Chart: GI ; At1g22770
| Category | GI At1g22770 |
|---|---|
### Chart
| Category | Bra024536 |
|---|---|
### Chart
| Category | Bra007774 | Bra034284 |
|---|---|---|
### Chart
| Category | Bra024536 |
|---|---|
### Chart: ELF3 ; At2g25930
| Category | ELF3 At2g25930 |
|---|---|
### Chart
| Category | Bra007774 | Bra034284 |
|---|---|---|G
H

## Slide 6
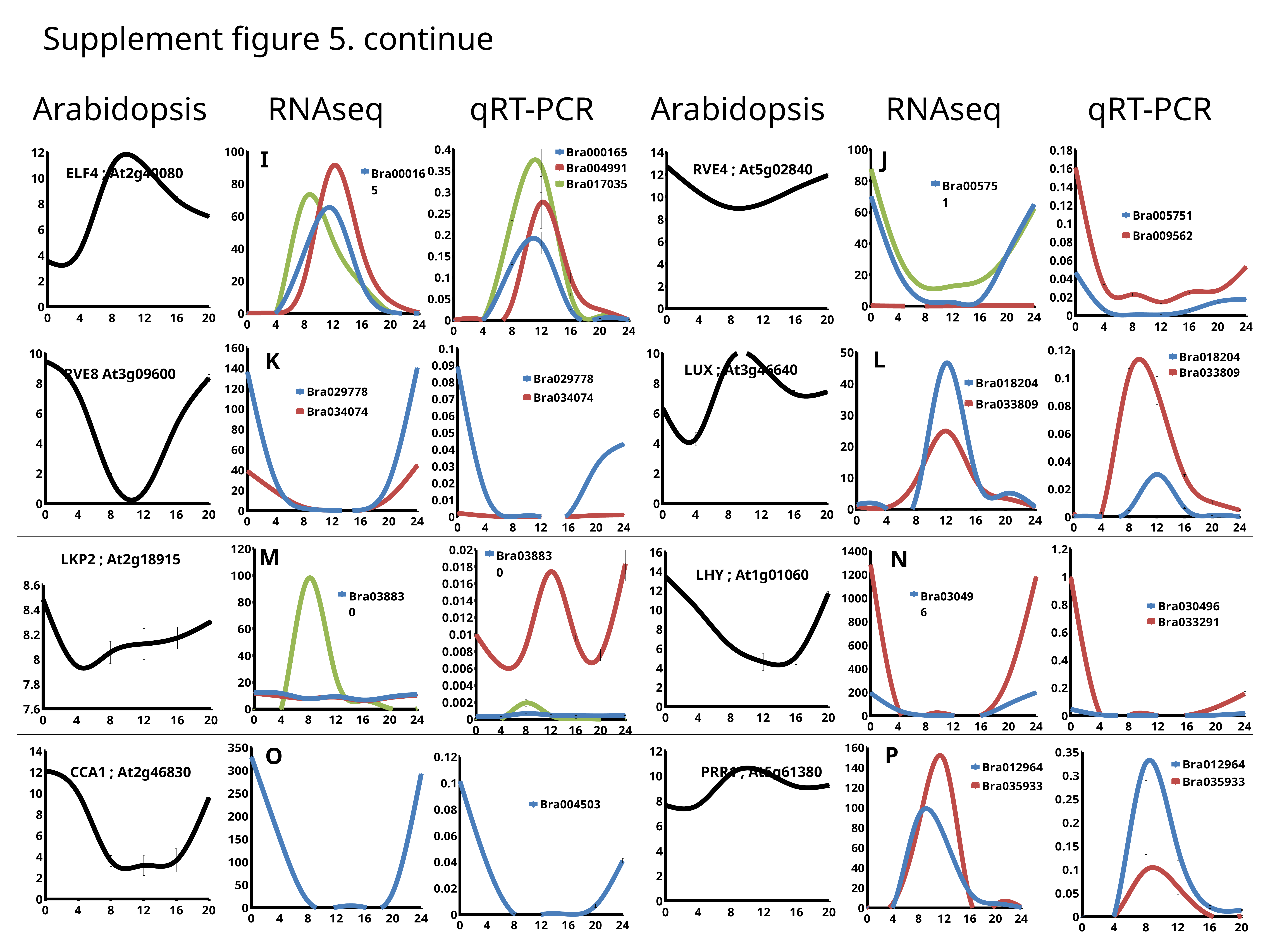

Supplement figure 5. continue
| Arabidopsis | RNAseq | qRT-PCR | Arabidopsis | RNAseq | qRT-PCR |
| --- | --- | --- | --- | --- | --- |
| | | | | | |
| | | | | | |
| | | | | | |
| | | | | | |
### Chart: ELF4 ; At2g40080
| Category | ELF4 At2g40080 |
|---|---|
### Chart
| Category | Bra000165 | Bra004991 | Bra017035 |
|---|---|---|---|
### Chart
| Category | Bra005751 | Bra005754 | Bra009562 |
|---|---|---|---|
### Chart
| Category | Bra005751 | Bra009562 |
|---|---|---|
### Chart
| Category | Bra000165 | Bra004991 | Bra017035 |
|---|---|---|---|
### Chart: RVE4 ; At5g02840
| Category | RVE4 At5g02840 |
|---|---|J
I
### Chart:
| Category | RVE8 At3g09600 |
|---|---|
### Chart
| Category | Bra029778 | Bra034074 |
|---|---|---|
### Chart: LUX ; At3g46640
| Category | LUX At3g46640 |
|---|---|
### Chart
| Category | Bra029778 | Bra034074 |
|---|---|---|
### Chart
| Category | Bra018204 | Bra033809 |
|---|---|---|
### Chart
| Category | Bra018204 | Bra033809 |
|---|---|---|L
K
### Chart: LKP2 ; At2g18915
| Category | LKP2 At2g18915 |
|---|---|
### Chart
| Category | Bra038830 | Bra038831 | Bra038832 |
|---|---|---|---|
### Chart
| Category | Bra038830 | Bra038831 | Bra038832 |
|---|---|---|---|
### Chart
| Category | Bra030496 | Bra033291 |
|---|---|---|
### Chart: LHY ; At1g01060
| Category | LHY At1g01060 |
|---|---|M
### Chart
| Category | Bra030496 | Bra033291 |
|---|---|---|N
### Chart: CCA1 ; At2g46830
| Category | CCA1 At2g46830 |
|---|---|
### Chart
| Category | Bra004503 |
|---|---|
### Chart: PRR1 ; At5g61380
| Category | PRR1 At5g61380 |
|---|---|
### Chart
| Category | Bra012964 | Bra035933 |
|---|---|---|P
O
### Chart
| Category | Bra012964 | Bra035933 |
|---|---|---|
### Chart
| Category | Bra004503 |
|---|---|

## Slide 7
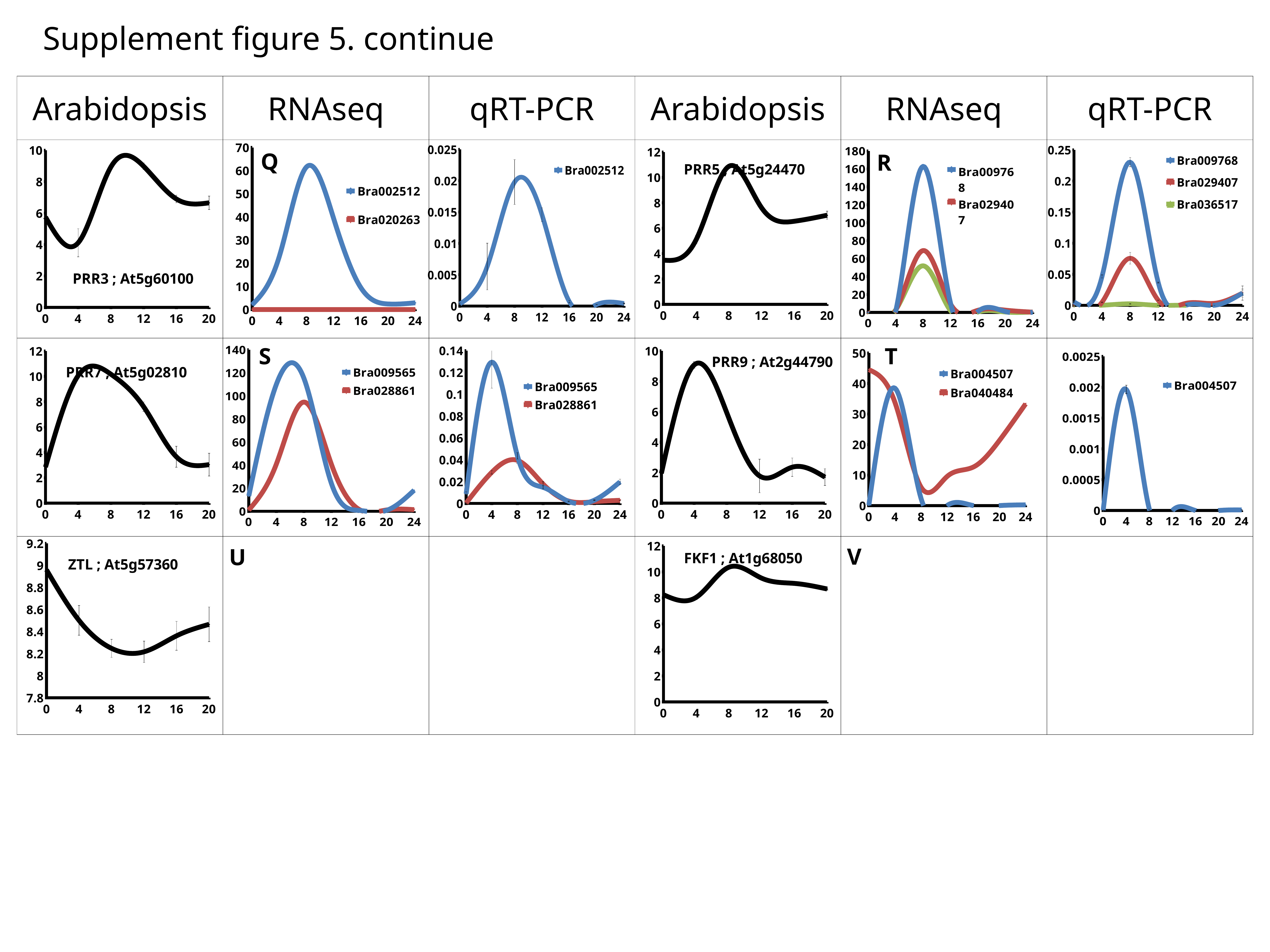

Supplement figure 5. continue
| Arabidopsis | RNAseq | qRT-PCR | Arabidopsis | RNAseq | qRT-PCR |
| --- | --- | --- | --- | --- | --- |
| | | | | | |
| | | | | | |
| | | | | | |
### Chart
| Category | Bra002512 | Bra020263 |
|---|---|---|
### Chart: PRR3 ; At5g60100
| Category | PRR3 At5g60100 |
|---|---|
### Chart
| Category | Bra002512 |
|---|---|
### Chart: PRR5 ; At5g24470
| Category | PRR5 At5g24470 |
|---|---|
### Chart
| Category | Bra009768 | Bra029407 | Bra036517 |
|---|---|---|---|
### Chart
| Category | Bra009768 | Bra029407 | Bra036517 |
|---|---|---|---|Q
R
### Chart
| Category | Bra009565 | Bra028861 |
|---|---|---|T
### Chart: PRR7 ; At5g02810
| Category | PRR7 At5g02810 |
|---|---|
### Chart
| Category | Bra009565 | Bra028861 |
|---|---|---|S
### Chart: PRR9 ; At2g44790
| Category | PRR9 At2g46790 |
|---|---|
### Chart
| Category | Bra004507 | Bra040484 |
|---|---|---|
### Chart
| Category | Bra004507 |
|---|---|
### Chart: ZTL ; At5g57360
| Category | ZTL At5g57360 |
|---|---|
### Chart: FKF1 ; At1g68050
| Category | FKF1 At1g68050 |
|---|---|V
U

## Slide 8
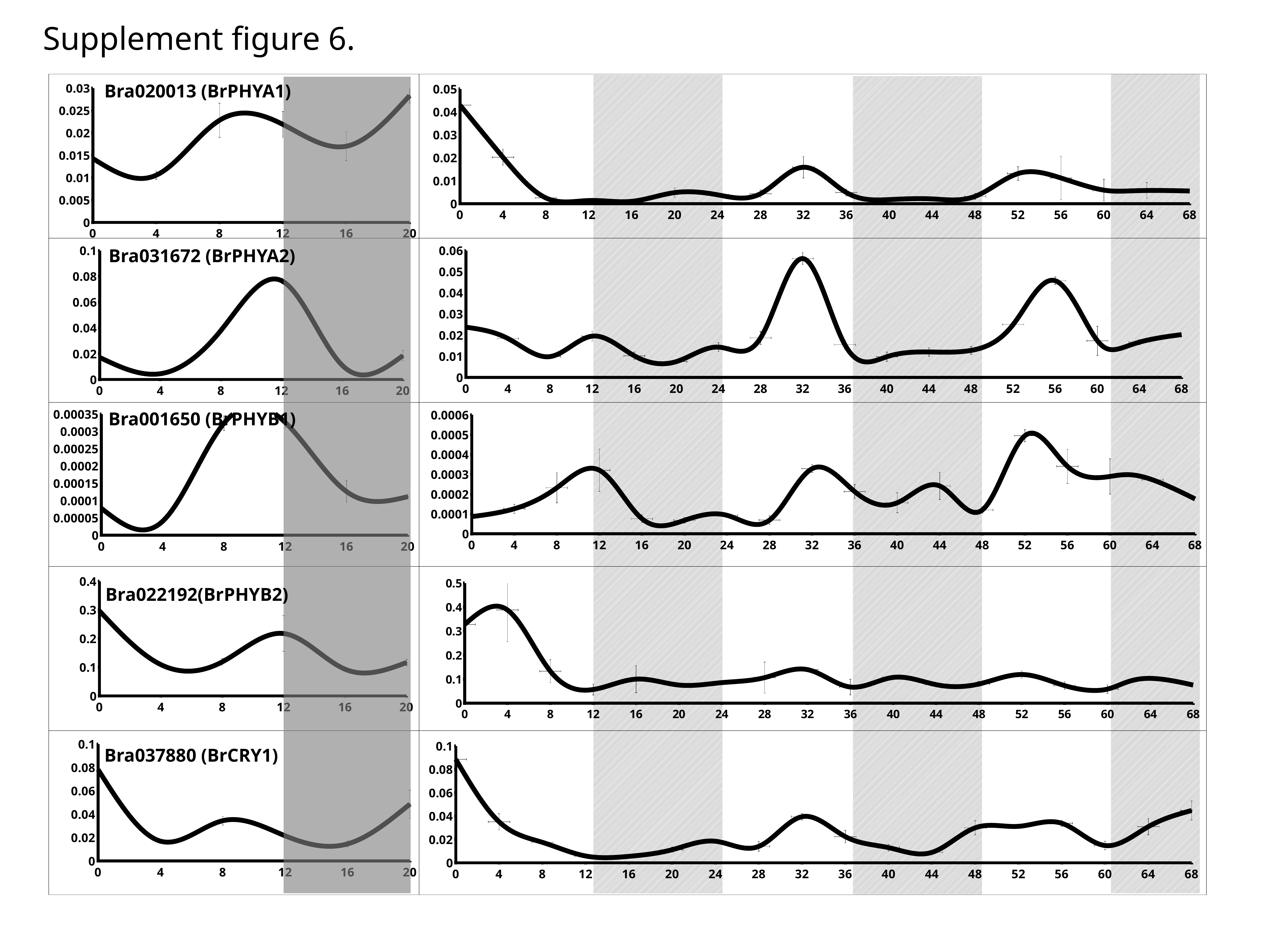

Supplement figure 6.
| | |
| --- | --- |
| | |
| | |
| | |
| | |
### Chart
| Category | |
|---|---|
Bra020013 (BrPHYA1)
### Chart
| Category | |
|---|---|
### Chart
| Category | |
|---|---|
### Chart
| Category | |
|---|---|Bra031672 (BrPHYA2)
### Chart
| Category | |
|---|---|
### Chart
| Category | |
|---|---|Bra001650 (BrPHYB1)
### Chart
| Category | |
|---|---|
### Chart
| Category | |
|---|---|Bra022192(BrPHYB2)
### Chart
| Category | |
|---|---|
### Chart
| Category | |
|---|---|Bra037880 (BrCRY1)

## Slide 9
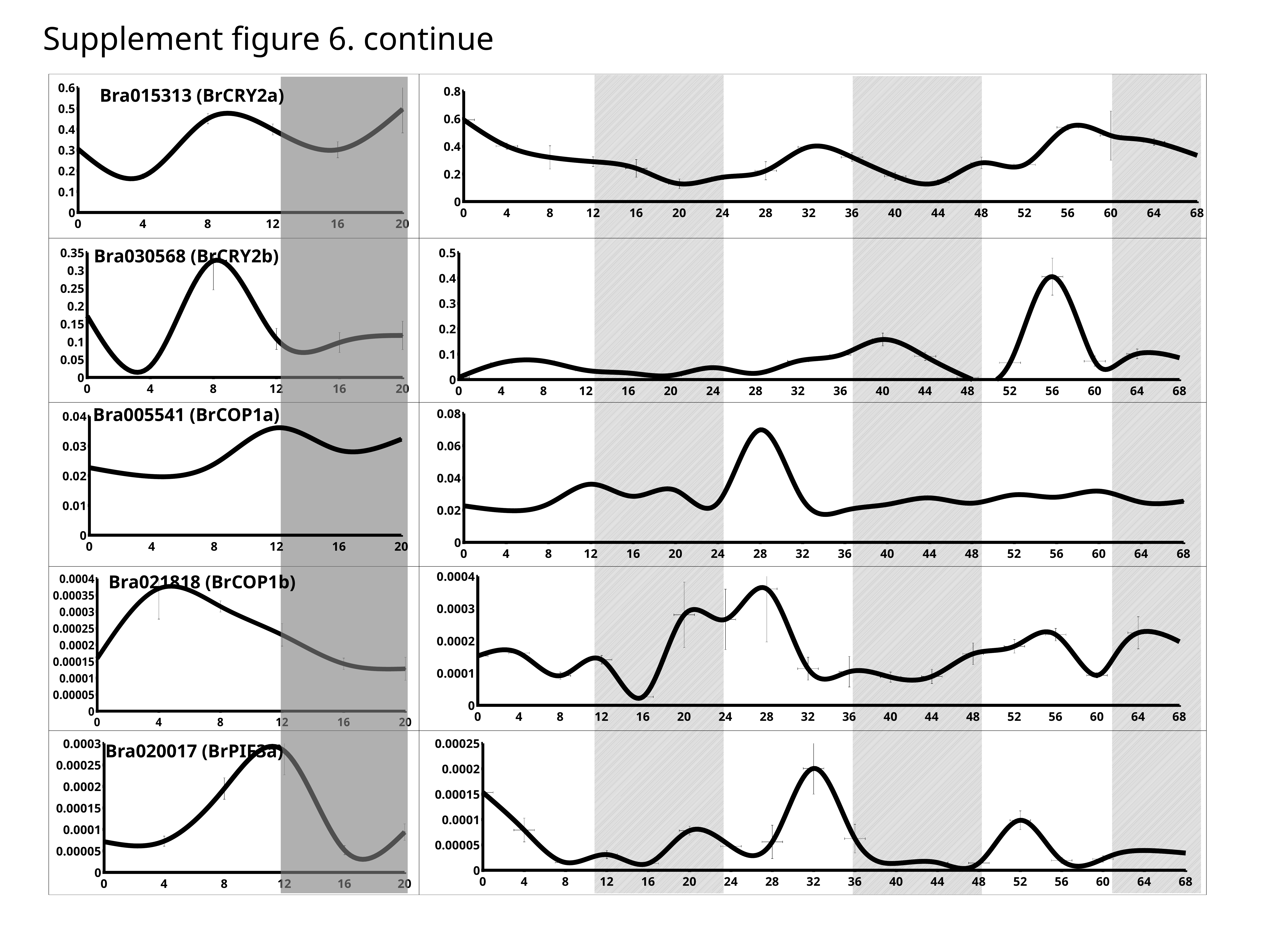

Supplement figure 6. continue
| | |
| --- | --- |
| | |
| | |
| | |
| | |
### Chart
| Category | |
|---|---|
### Chart
| Category | |
|---|---|Bra015313 (BrCRY2a)
### Chart
| Category | |
|---|---|
### Chart
| Category | |
|---|---|Bra030568 (BrCRY2b)
Bra005541 (BrCOP1a)
### Chart
| Category | |
|---|---|
### Chart
| Category | |
|---|---|
### Chart
| Category | |
|---|---|
### Chart
| Category | |
|---|---|Bra021818 (BrCOP1b)
### Chart
| Category | |
|---|---|
### Chart
| Category | |
|---|---|Bra020017 (BrPIF3a)

## Slide 10
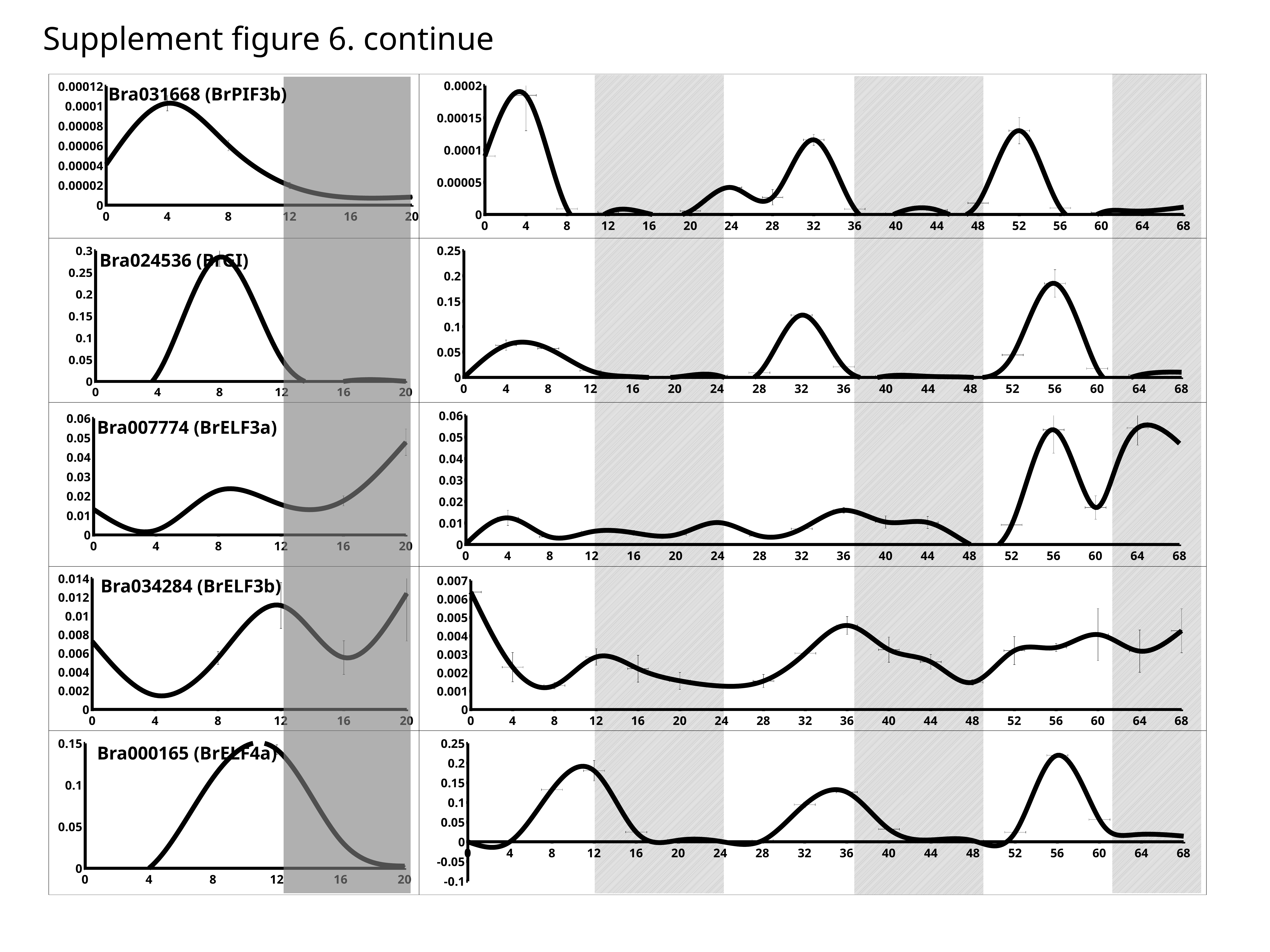

Supplement figure 6. continue
| | |
| --- | --- |
| | |
| | |
| | |
| | |
### Chart
| Category | |
|---|---|
### Chart
| Category | |
|---|---|
Bra031668 (BrPIF3b)
### Chart
| Category | |
|---|---|
### Chart
| Category | |
|---|---|Bra024536 (BrGI)
### Chart
| Category | |
|---|---|
### Chart
| Category | |
|---|---|Bra007774 (BrELF3a)
### Chart
| Category | |
|---|---|
### Chart
| Category | |
|---|---|Bra034284 (BrELF3b)
### Chart
| Category | |
|---|---|
### Chart
| Category | |
|---|---|Bra000165 (BrELF4a)

## Slide 11
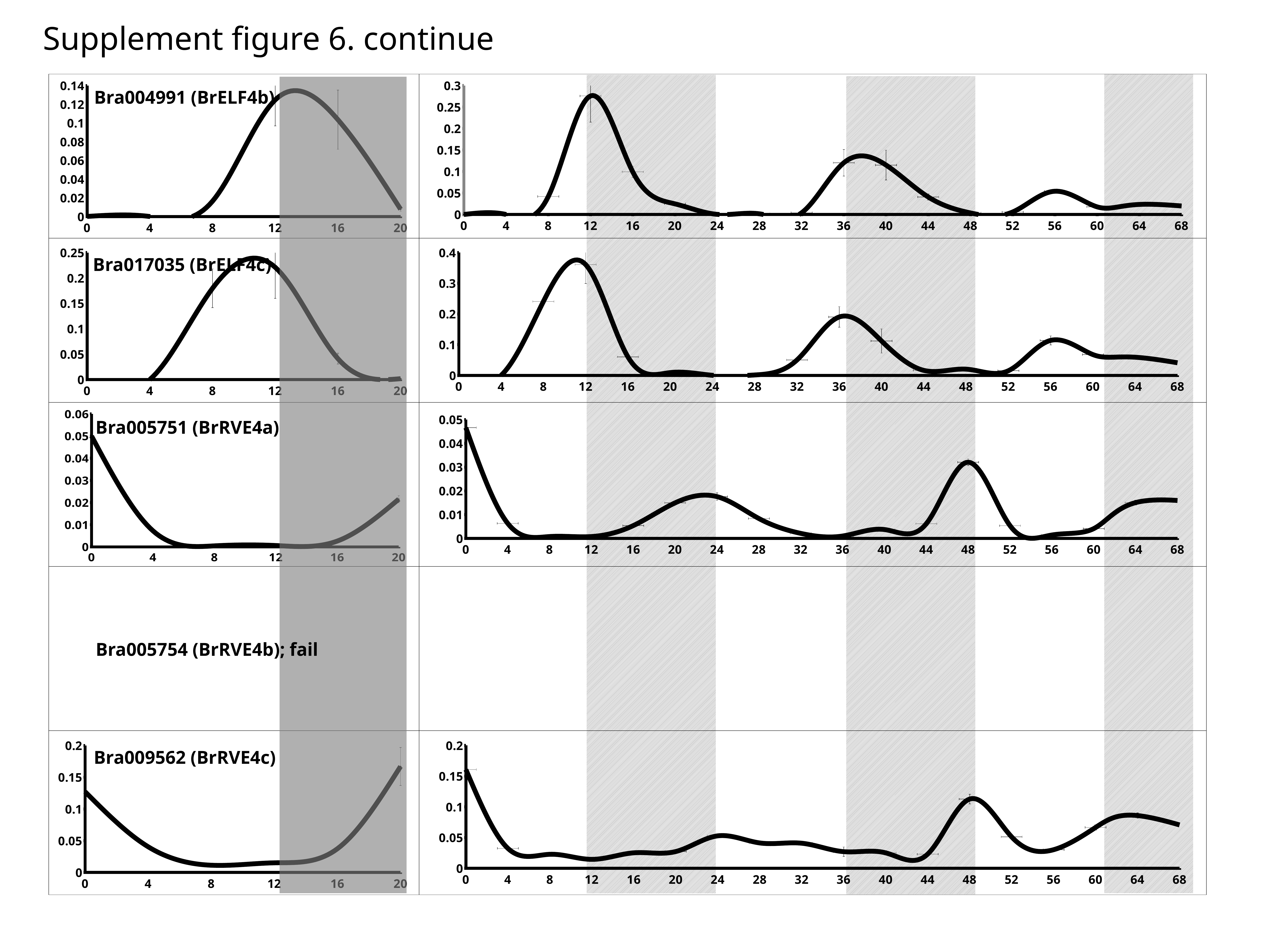

Supplement figure 6. continue
| | |
| --- | --- |
| | |
| | |
| | |
| | |
### Chart
| Category | |
|---|---|
### Chart
| Category | |
|---|---|
Bra004991 (BrELF4b)
### Chart
| Category | |
|---|---|
### Chart
| Category | |
|---|---|Bra017035 (BrELF4c)
### Chart
| Category | |
|---|---|
### Chart
| Category | |
|---|---|Bra005751 (BrRVE4a)
Bra005754 (BrRVE4b); fail
### Chart
| Category | |
|---|---|
### Chart
| Category | |
|---|---|Bra009562 (BrRVE4c)

## Slide 12
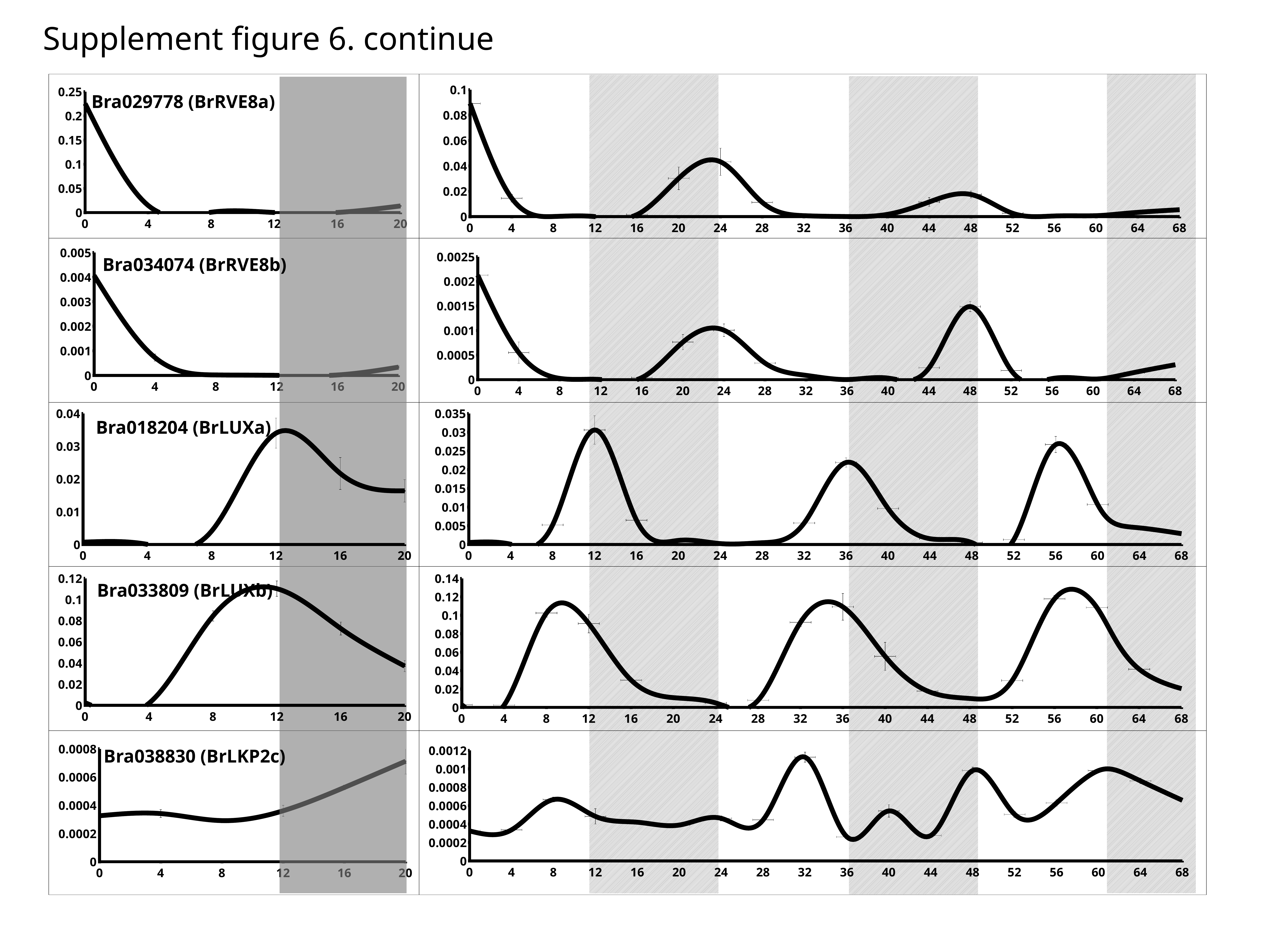

Supplement figure 6. continue
| | |
| --- | --- |
| | |
| | |
| | |
| | |
### Chart
| Category | |
|---|---|
### Chart
| Category | |
|---|---|Bra029778 (BrRVE8a)
### Chart
| Category | |
|---|---|
### Chart
| Category | |
|---|---|Bra034074 (BrRVE8b)
### Chart
| Category | |
|---|---|
### Chart
| Category | |
|---|---|Bra018204 (BrLUXa)
### Chart
| Category | |
|---|---|
### Chart
| Category | |
|---|---|Bra033809 (BrLUXb)
### Chart
| Category | |
|---|---|
### Chart
| Category | |
|---|---|Bra038830 (BrLKP2c)

## Slide 13
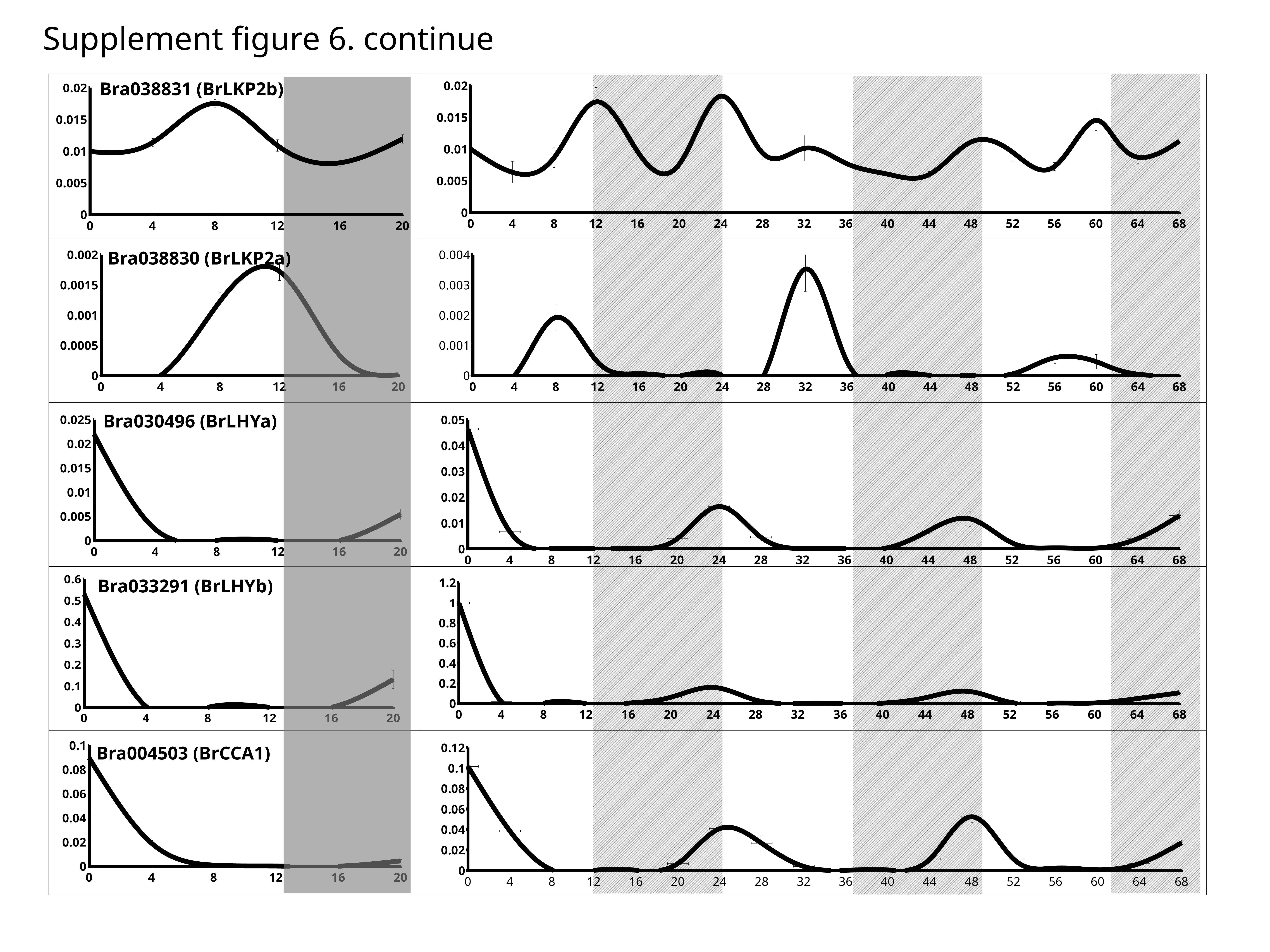

Supplement figure 6. continue
| | |
| --- | --- |
| | |
| | |
| | |
| | |
### Chart
| Category | |
|---|---|
### Chart
| Category | |
|---|---|Bra038831 (BrLKP2b)
### Chart
| Category | |
|---|---|
### Chart
| Category | |
|---|---|Bra038830 (BrLKP2a)
### Chart
| Category | |
|---|---|Bra030496 (BrLHYa)
### Chart
| Category | |
|---|---|
### Chart
| Category | |
|---|---|
### Chart
| Category | |
|---|---|Bra033291 (BrLHYb)
### Chart
| Category | |
|---|---|
### Chart
| Category | |
|---|---|Bra004503 (BrCCA1)

## Slide 14
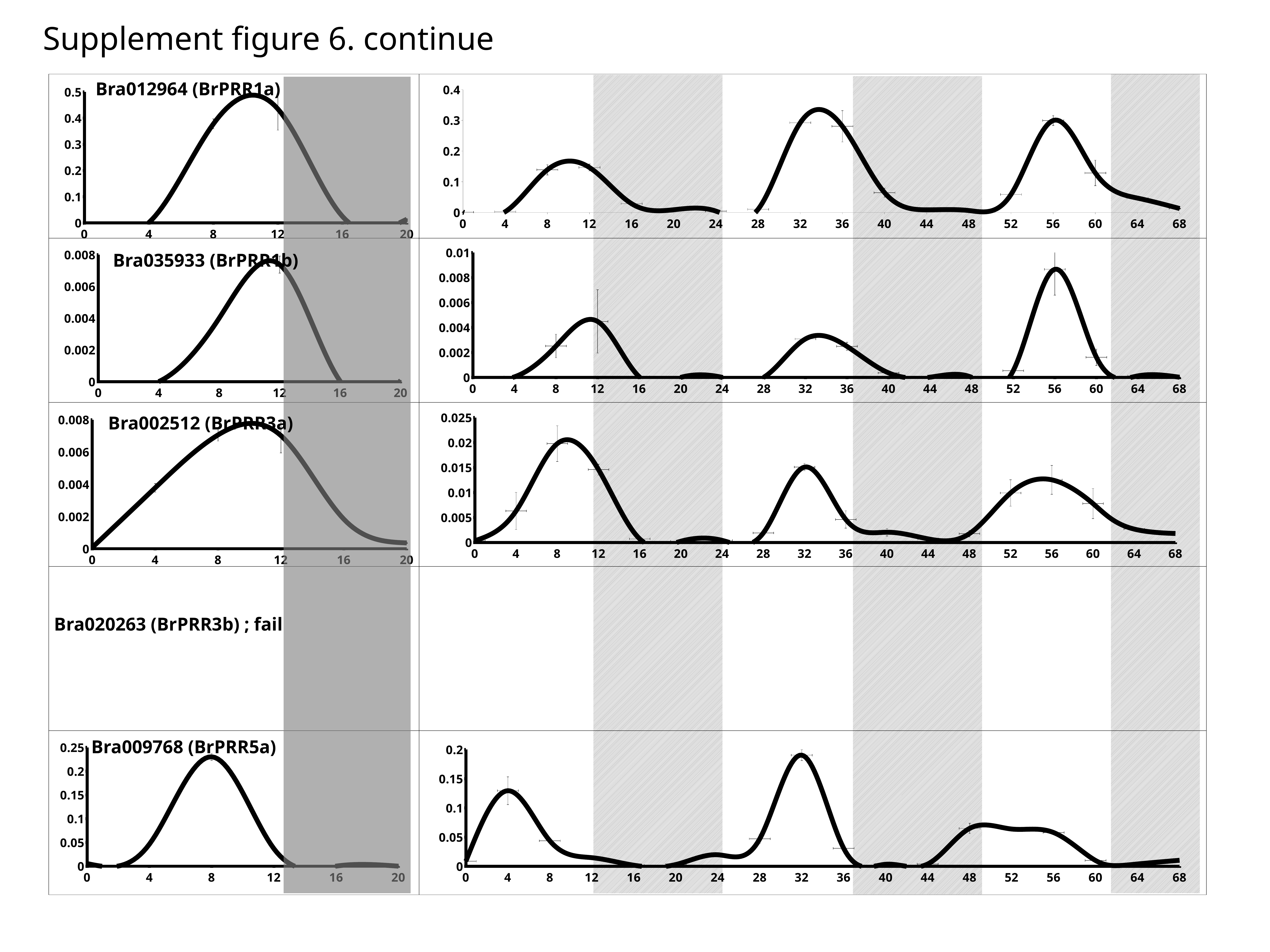

Supplement figure 6. continue
| | |
| --- | --- |
| | |
| | |
| | |
| | |
Bra012964 (BrPRR1a)
### Chart
| Category | |
|---|---|
### Chart
| Category | |
|---|---|
### Chart
| Category | |
|---|---|
### Chart
| Category | |
|---|---|Bra035933 (BrPRR1b)
### Chart
| Category | |
|---|---|
### Chart
| Category | |
|---|---|Bra002512 (BrPRR3a)
Bra020263 (BrPRR3b) ; fail
Bra009768 (BrPRR5a)
### Chart
| Category | |
|---|---|
### Chart
| Category | |
|---|---|

## Slide 15
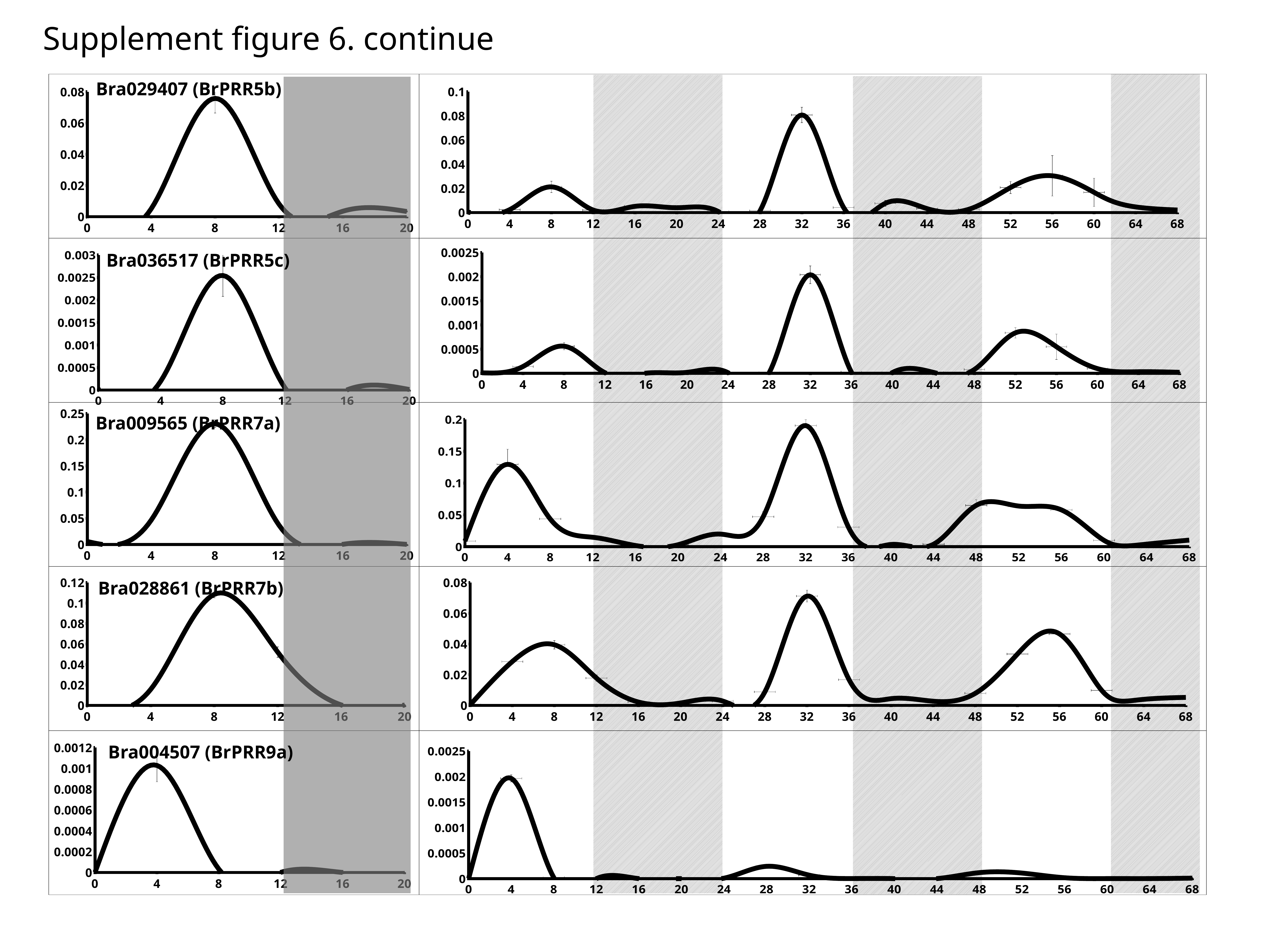

Supplement figure 6. continue
| | |
| --- | --- |
| | |
| | |
| | |
| | |
Bra029407 (BrPRR5b)
### Chart
| Category | |
|---|---|
### Chart
| Category | |
|---|---|
### Chart
| Category | |
|---|---|
### Chart
| Category | |
|---|---|Bra036517 (BrPRR5c)
### Chart
| Category | Aver |
|---|---|
### Chart
| Category | |
|---|---|Bra009565 (BrPRR7a)
### Chart
| Category | |
|---|---|
### Chart
| Category | |
|---|---|Bra028861 (BrPRR7b)
### Chart
| Category | |
|---|---|
### Chart
| Category | |
|---|---|Bra004507 (BrPRR9a)

## Slide 16
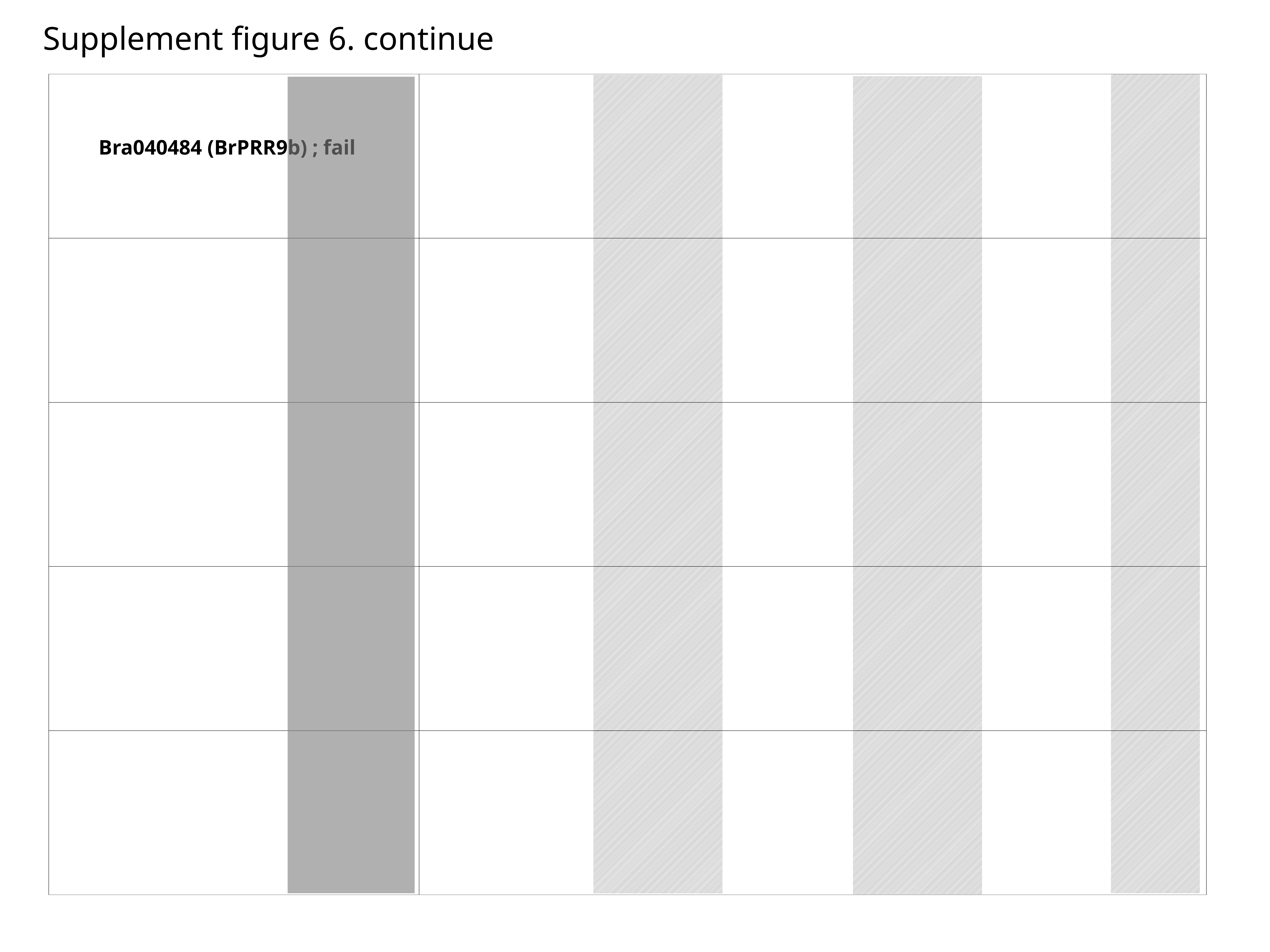

Supplement figure 6. continue
| | |
| --- | --- |
| | |
| | |
| | |
| | |
Bra040484 (BrPRR9b) ; fail
