# Supplementary material for: Transcriptome Analysis of Diurnal Gene Expression in Chinese Cabbage
Source: Genes (Basel). 2019 Feb 11;10(2):130. doi: 10.3390/genes10020130 (PMC6409912; doi:10.3390/genes10020130)
Supplement: Supplementary file 1 [file genes-10-00130-s001.zip › Supplement Table1.docx]

**Supplementary Table S1. PCR primers used in this work.**

| Brassica ID | Gene symbol in Arabidopsis | Polarity | Amplicon length | Primer sequence |
| --- | --- | --- | --- | --- |
| Bra020013 | PHYA | Forward | 229 | 5`-CCGCGTCACCGGACCCGT |
|  |  | Reverse |  | 5`-CCGCGTCACCGGACCCGT |
| Bra031672 |  | Forward | 235 | 5`-AGTGCGTGTAACTACCCCAGC |
|  |  | Reverse |  | 5`-AACGCCTAGAACGGGGCTCTCG |
| Bra001650 | PHYB | Forward | 199 | 5`-TAATCCGAATGGCGTGTCCAG |
|  |  | Reverse |  | 5`-ATTCTTGAACTCCCCGTTCC |
| Bra022192 |  | Forward | 248 | 5`-ATGGCTGATGGATTCTACGC |
|  |  | Reverse |  | 5`-GACAAAGGGCGTTTCATAGG |
| Bra037880 | CRY1 | Forward | 227 | 5`-CGCAGATGTGGCAACTAGAA |
|  |  | Reverse |  | 5`-CGACTCTTGGTCTTCTTCAGG |
| Bra015313 | CRY2 | Forward | 200 | 5`-TGGAGTGGAGCTCGGTGC |
|  |  | Reverse |  | 5`-CAGAGGGGCAAAGAAGACCTGG |
| Bra030568 |  | Forward | 148 | 5`-CTGCTAATACAGTTAAAGAACAT |
|  |  | Reverse |  | 5`-TCATTGAATCCTCTAAGTTT |
| Bra005541 | COP1 | Forward |  | 5`- |
|  |  | Reverse |  | 5`- |
| Bra021818 |  | Forward | 240 | 5`-CGGTTCGGACATTTAGAGGA |
|  |  | Reverse |  | 5`-CAGTCAGCATCGTGGGACTA |
| Bra020017 | PIF3 | Forward | 247 | 5`-TTTCTCCCGTTACCGTCAACG |
|  |  | Reverse |  | 5`-ACATTATTAGTCTTAGCCAA |
| Bra031668 |  | Forward | 271 | 5`-TCCTCCTGCTGCTGCTTCTTCG |
|  |  | Reverse |  | 5`-CATAGCAGAGGCAACAGA |
| Bra024536 | GI | Forward | 245 | 5`-ACGTCCACGTCACGTAATGA |
|  |  | Reverse |  | 5`-AGCGAAACAACGGAGAAAGA |
| Bra007774 | ELF3 | Forward | 280 | 5`-TCTGCAACAAGAGATAAATAATG |
|  |  | Reverse |  | 5`-ATAGCTTTTAGCAGCAACT |
| Bra034284 |  | Forward | 226 | 5`-GTGGGGTGGAGAGAAACTTG |
|  |  | Reverse |  | 5`-AAAGTGGTGCGTTTTTCACC |
| Bra000165 | ELF4 | Forward | 158 | 5`-ATCATCAATCAAAAATGGCT |
|  |  | Reverse |  | 5`-ACCACCACCGTCGTGTCGGTTA |
| Bra004991 |  | Forward | 185 | 5`-ATAGATCACTTATCCAGCA |
|  |  | Reverse |  | 5`-ACCACCGTCGCGACCGTTCTT |
| Bra017035 |  | Forward | 255 | 5`-GAGGAGAAGATCCGGCGATGT |
|  |  | Reverse |  | 5`-CACCCTCGTGTCCGTTCTT |
| Bra005751 | RVE4 | Forward | 115 | \| 5`-GTGGTTTCTGAAGAAAGACCGG \| \| --- \| |
|  |  | Reverse |  | 5`-AACTCTGTCTGGACTTGGA |
| Bra005754 |  | Forward | 149 | 5`-ACTGTAATATAGCTAAAAAGTT |
|  |  | Reverse |  | 5`-CTGAACCTTCAAAAAATAT |
| Bra009562 |  | Forward | 176 | 5`-TCGCTGAAGAAGAAAGACCGAT |
|  |  | Reverse |  | 5`-CCAGTCACGATCAAACAATTG |
| Bra029778 | RVE8 | Forward | 216 | 5`-GAAGATTTCGTGGGTTCCAA |
|  |  | Reverse |  | 5`-ACCAGCCATGTCGCTATTTC |
| Bra034074 |  | Forward | 201 | 5`-GACGCATCAATGCTGCTAAA |
|  |  | Reverse |  | 5`-ATGAAGCACTGGAGGCTGTT |
| Bra018204 | LUX | Forward | 240 | 5`-CAAGATATCGGAGGCGGTAA |
|  |  | Reverse |  | 5`-CACTTCTACCCCCAACAGGA |
| Bra033809 |  | Forward | 165 | 5`-GGCGTCGCTCTCGTACTCTCTTGT |
|  |  | Reverse |  | 5`-AGGTTGGTGGACGAAGAGGT |
| Bra038830 | LKP2 | Forward | 125 | 5`-CGATGCTGATATCGATCTC |
|  |  | Reverse |  | 5`-TCCCACATAGTCCACGAGAA |
| Bra038831 |  | Forward | 181 | 5`-TGCAGAAATCGATCTCGGCCCC |
|  |  | Reverse |  | 5`-AGGGGGAGTCAGCTTTGACAA |
| Bra038832 |  | Forward | 112 | 5`-AAATCAGATGGAGTGGGATAG |
|  |  | Reverse |  | 5`-GGTGACCACGAACCCAAAC |
| Bra030496 | LHY | Forward | 254 | 5`-AGACATTCATGGAATCTTCTG |
|  |  | Reverse |  | 5`-GCGGTAATCATGCTGGAAAG |
| Bra033291 |  | Forward | 228 | 5`-CTGTATGCGCTCCTCCTGCT |
|  |  | Reverse |  | 5`-TTGTCTTTGAGCCAGCGTTG |
| Bra004503 | CCA1 | Forward | 184 | 5`-TCTCTGTCACATGCTCCTCCTT |
|  |  | Reverse |  | 5`-CGGCTAAGTTCCCTTGTGG |
| Bra012964 | PRR1 | Forward | 227 | 5`-GTTCCGTTTGTGCTGGCGAT |
|  |  | Reverse |  | 5`-GCCATTTCCATTGACAGTA |
| Bra035933 |  | Forward | 165 | 5`-ACCAACTGTCAAGATCCTC |
|  |  | Reverse |  | 5`-GGCGCCGCCATTCTGTA |
| Bra002512 | PRR3 | Forward | 260 | 5`-CAAGCAGTGACAATCCT |
|  |  | Reverse |  | 5`-TCAGTCTTCTTTACCAATTTTGC |
| Bra020263 |  | Forward | 254 | 5`-GCAGTATTGACAATCCA |
|  |  | Reverse |  | 5`-CTATACCATCTTCGTTGACTC |
| Bra009768 | PRR5 | Forward | 217 | 5`-TTAGCAACCAGATGAATT |
|  |  | Reverse |  | 5`-TCCATATCCATGGAAC |
| Bra029407 |  | Forward | 233 | 5`-ATCTTCAGGAGAAAAGC |
|  |  | Reverse |  | 5`-AAGAATTCATCTGAGATG |
| Bra036517 |  | Forward | 178 | 5`-AGTGTTAGGATAGAGCTTG |
|  |  | Reverse |  | 5`-TGGTTACCACAATGT |
| Bra009565 | PRR7 | Forward | 197 | 5`-ATAAGCTTTCTGGCGGGAAC |
|  |  | Reverse |  | 5`-AGGCTTTGTGAAAGCGTTGT |
| Bra028861 |  | Forward | 162 | 5`-TAAAACCACTGGCATCGTGA |
|  |  | Reverse |  | 5`-TGGCTAGACAACGCTTTGAA |
| Bra004507 | PRR9 | Forward | 213 | 5`-TAGGCATTACATTAACTTG |
|  |  | Reverse |  | 5`-GGTTACGGAAGCTTAACTCATTT |
| Bra040484 |  | Forward | 226 | 5`-GGATGTCAAACCGCTGTTTG |
|  |  | Reverse |  | 5`-TCTTGAGAAGGCTGATGC |
